# Metabolic Nanomedicine Reprograms Mesenchymal Stem Cells for Durable Exosome‐Mediated Immunomodulation

**DOI:** 10.1002/exp2.70249

**Published:** 2026-11-10

**Authors:** Jinfeng Chen, Kanghua Zeng, Jinhong Chao, Xiangna Zhang, Zheng Zhou, Xiang Li, Wancheng Liu, Xiahong Xie, Luyao Wang, Mingzhou Ye

**Affiliations:** ^1^ Division of Nanobiomedicine, Suzhou Institute of Nano‐Tech and Nano‐Bionics (SINANO) Chinese Academy of Sciences Suzhou China; ^2^ Jiangsu Key Laboratory of Organoid Engineering and Precision Medicine Suzhou China; ^3^ University of Science and Technology of China Hefei China; ^4^ Department of Clinical Laboratory Qilu Hospital of Shandong University Jinan China

**Keywords:** exosome, inflammatory regulation, mesenchymal stem cells, microRNA, NAD^+^

## Abstract

Mesenchymal stem cells (MSCs) are widely recognized for their immunomodulatory potential, yet their clinical efficacy remains inconsistent due to poor persistence and functional instability under prolonged inflammatory stress. Here we identify NAD^+^ depletion as a central driver of MSCs exhaustion, leading to metabolic collapse, programmed cell death, and loss of immunoregulatory activity. Direct restoration is nontrivial, because NAD^+^ is membrane impermeable and extracellularly unstable. To overcome this barrier, we developed a nanoparticle system for efficient intracellular delivery of NAD^+^, enabling sustained metabolic support even under hostile inflammatory conditions. NAD^+^ restoration markedly improved MSCs survival, preserved their ability to suppress T cell proliferation, and enhanced therapeutic efficacy in murine colitis. Mechanistically, NAD^+^ supplementation did not increase anti‐inflammatory mediator release but instead reprogrammed exosome function, enhancing secretion and selectively enriching key anti‐inflammatory microRNAs. This effect was mediated by an NAD^+^/α‐ketoglutarate/TET1‐SMPD3 axis involving promoter demethylation, exosome biogenesis, and microRNA sorting. Together, these findings uncover an epigenetic‐metabolic circuit that links NAD^+^ availability to exosome‐mediated immune regulation, providing a mechanistic foundation for developing metabolically fortified MSCs platforms with enhanced persistence and translational potential in inflammatory disease.

## Graphical abstract

Prolonged inflammatory stress depletes NAD^+^ in mesenchymal stem cells, driving functional exhaustion and weakening their survival and immune‐regulating activity. We developed an NAD^+^‐delivering nanomedicine that restores cellular metabolism and improves therapeutic efficacy in colitis. The treatment also enhances exosome production and enriches anti‐inflammatory microRNAs through a TET1‐SMPD3 pathway, enabling more durable immune regulation.

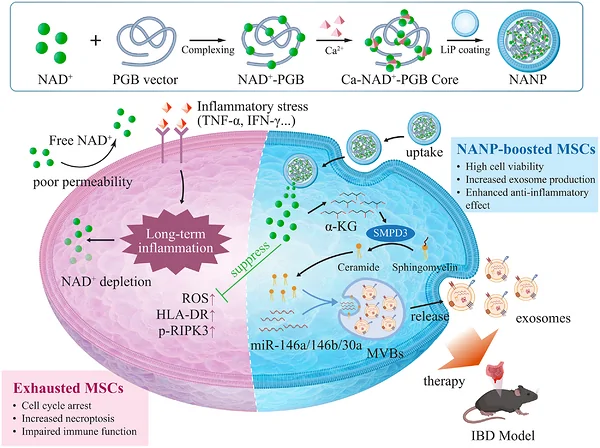

## 1 Introduction

Mesenchymal stem cells (MSCs) have long attracted interest for their capacity to differentiate, modulate immunity, and support tissue repair [1]. More than 1500 clinical trials explore their use in disorders such as graft‐versus‐host disease, inflammatory bowel disease (IBD), and rheumatoid arthritis [2]. However, despite promising translational progress, therapeutic efficacy remains inconsistent, largely due to the harsh inflammatory milieu in vivo, where MSCs exhibit significant vulnerability to apoptosis triggered by hypoxia, oxidative stress, and chronic cytokine exposure [3, 4]. Notably, fewer than 28% of intravenously infused MSCs survive at 24 h post‐administration, and viability drops below 1% by Day 7 [5, 6].

Escalating MSCs dosages to counter poor engraftment, raises cost and safety concerns, prompting exploration of approaches, such as inflammatory preconditioning and biomaterial‐assisted delivery [7]. These methods have improved MSCs survival in some settings but also bring challenges: extensive in vitro manipulation may raise the risk of genetic instability, while synthetic scaffolds can hinder cell trafficking and complicate scalable production [8]. As a result, interest has shifted toward MSCs‐derived exosomes, 50–150 nm nanoscale vesicles that carry RNAs, lipids, and proteins that recapitulate much of their parent cells’ immunomodulatory activity [9]. However, exosomes administered systemically are rapidly cleared by phagocytes, with therapeutic effects typically lasting less than 48 h [10, 11]. A continued need exists for strategies that sustain in vivo exosome production and strengthen their anti‐inflammatory potency, ideally by harnessing the intrinsic capacity of MSCs to serve as a continuous source of bioactive vesicles within the host milieu.

NAD^+^ is capable of sustaining MSCs fitness in inflammatory environments and holds the potential to reprogram cellular metabolism toward greater immunomodulatory activity, positioning it as a key modulator of MSCs‐centered therapies. As a central metabolic coenzyme, NAD^+^ supports mitochondrial integrity, redox balance, and energy production, and recent studies further implicate it in controlling immune signaling and cell survival pathways [12, 13]. Inflammatory stress, characterized by surges of cytokines and reactive oxygen species (ROS), accelerates NAD^+^ consumption and impairs its synthesis, leading to profound intracellular depletion that compromises essential enzymatic processes and drives stem cell functional decline [14, 15, 16]. Preserving NAD^+^ availability may therefore represent a metabolic checkpoint to sustain MSCs fitness and therapeutic potency in hostile microenvironments.

However, efficient intracellular restoration of NAD^+^ in vivo is intrinsically challenging. Intact NAD^+^ is highly polar and lacks efficient transport into the cytosol, and extracellular NAD^+^ is readily consumed by ectoenzymes, reducing bioavailability. In parallel, precursor‐based augmentation depends on ATP‐intensive salvage steps such as NRK and NAMPT, which are compromised during inflammatory energy crisis and further outpaced by heightened NAD^+^ consumption. These constraints make precursor dosing unreliable in inflamed tissues and motivate a delivery system that bypasses membrane transport and salvage limitations.

Here, we demonstrated that prolonged inflammatory exposure drives irreversible NAD^+^ depletion in MSCs, leading to proliferation arrest, increased susceptibility to cell death, and loss of immunoregulatory function. To address this metabolic vulnerability, we developed a poly(β‐amino ester) polymer (PBAE) bearing alternating guanidine and phenylboronic acid groups (denoted as PGB) with high affinity for NAD^+^, enabling the creation of a nanomedicine that efficiently restores intracellular NAD^+^. Replenishment of NAD^+^ preserved MSCs viability under chronic inflammation, reversed functional exhaustion, and strengthened the anti‐inflammatory potency of their secreted exosomes. In a murine colitis model, NAD^+^‐fortified MSCs exhibited improved homing and persistence at inflamed sites and conferred superior therapeutic benefit compared with unmodified cells (Scheme 1). Mechanistic analyses revealed an NAD^+^/α‐ketoglutarate (α‐KG)/TET1‐SMPD3 epigenetic axis that governs selective loading of immunoregulatory microRNAs into exosomes, defining a metabolic–epigenetic circuit that can be harnessed to develop metabolically fortified MSCs therapies with improved durability and immunomodulatory efficacy.

**SCHEME 1:**
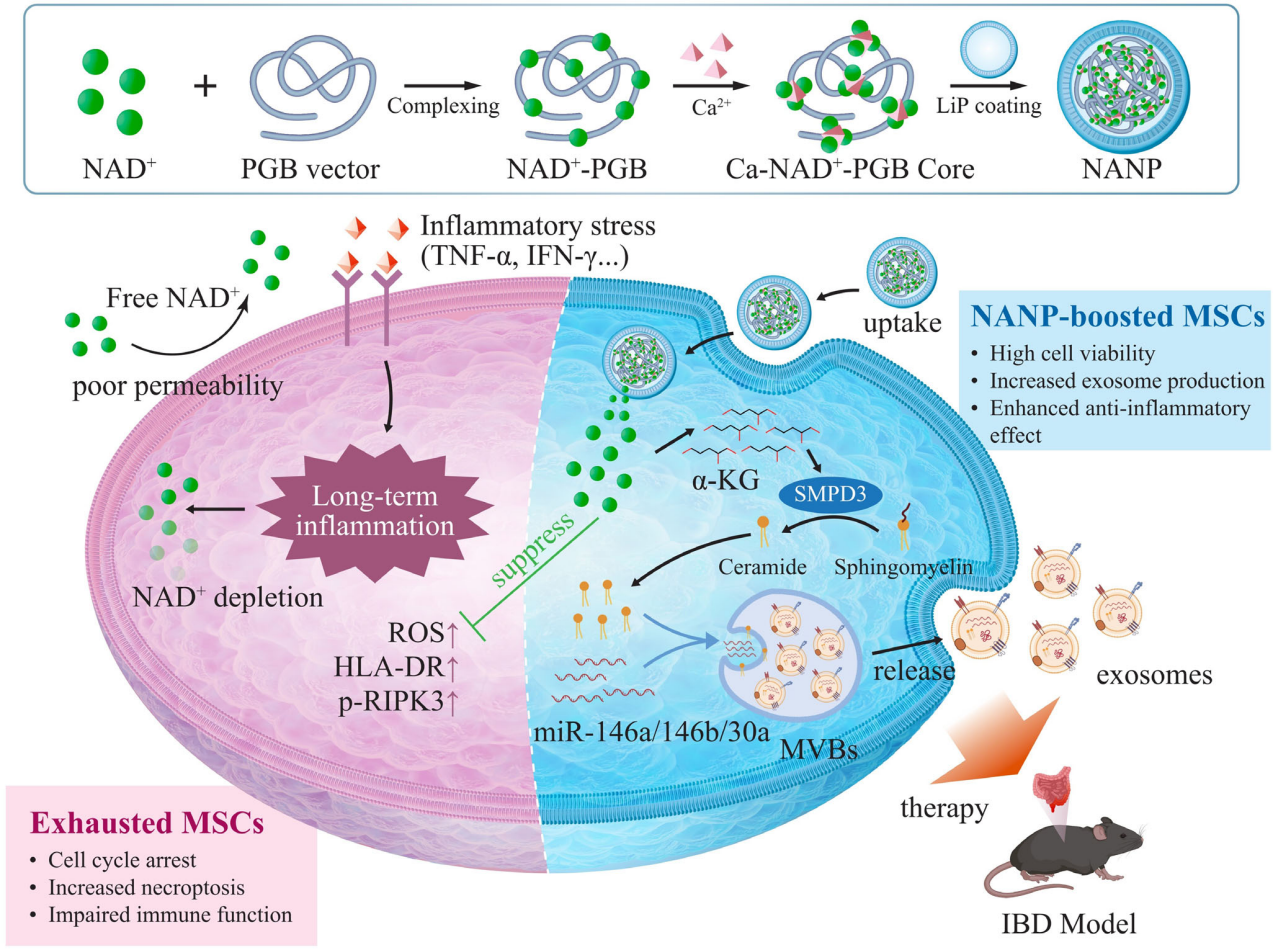
Schematic illustration of NANP‐primed MSCs and their paracrine effects on IBD. NANP pretreatment replenishes intracellular NAD^+^ in MSCs, preventing their exhaustion under inflammatory conditions, and upregulates α‐KG to promote SMPD3 expression, thereby enhancing exosome biogenesis and specific miRNA packaging. Upon systemic administration, these NANP‐boosted MSCs ameliorate IBD in mice primarily through the action of their secreted exosomes.

## 2 Results

### 2.1 Fabrication and Characterization of NAD^+^ Nanoparticles for Sustaining NAD^+^ Levels in MSCs Under Inflammatory Stress

Inflammatory cytokines are known to transiently augment the immunomodulatory properties of MSCs, forming the basis of preconditioning strategies to enhance therapeutic efficacy [17]. However, sustained inflammatory exposure triggers a paradoxical response: it initially enhances the immunosuppressive function while concurrently inducing apoptosis and metabolic stress, ultimately compromising MSCs viability and long‐term functionality [18, 19]. To investigate the fate of MSCs under inflammatory stress in vivo, we generated luciferase‐expressing MSCs (MSCs‐Luc) and validated their luciferase activity (Figure S1A,B). In a lipopolysaccharide (LPS)‐induced systemic inflammation model [20], intraperitoneally administered MSCs‐Luc exhibited a progressive decline in bioluminescence, indicating rapid loss of viability under inflammatory conditions (Figure S1C–E). In vitro, exposure to a combination treatment of IFN‐γ and TNF‐α (IT, 10 ng mL^−1^ each) had minimal impact on cell viability and apoptosis within the first 24 h, but led to pronounced growth arrest and a significant increase in apoptotic cells after 48 h of exposure (Figure 1A, Figure S1F,G).

**FIGURE 1:**
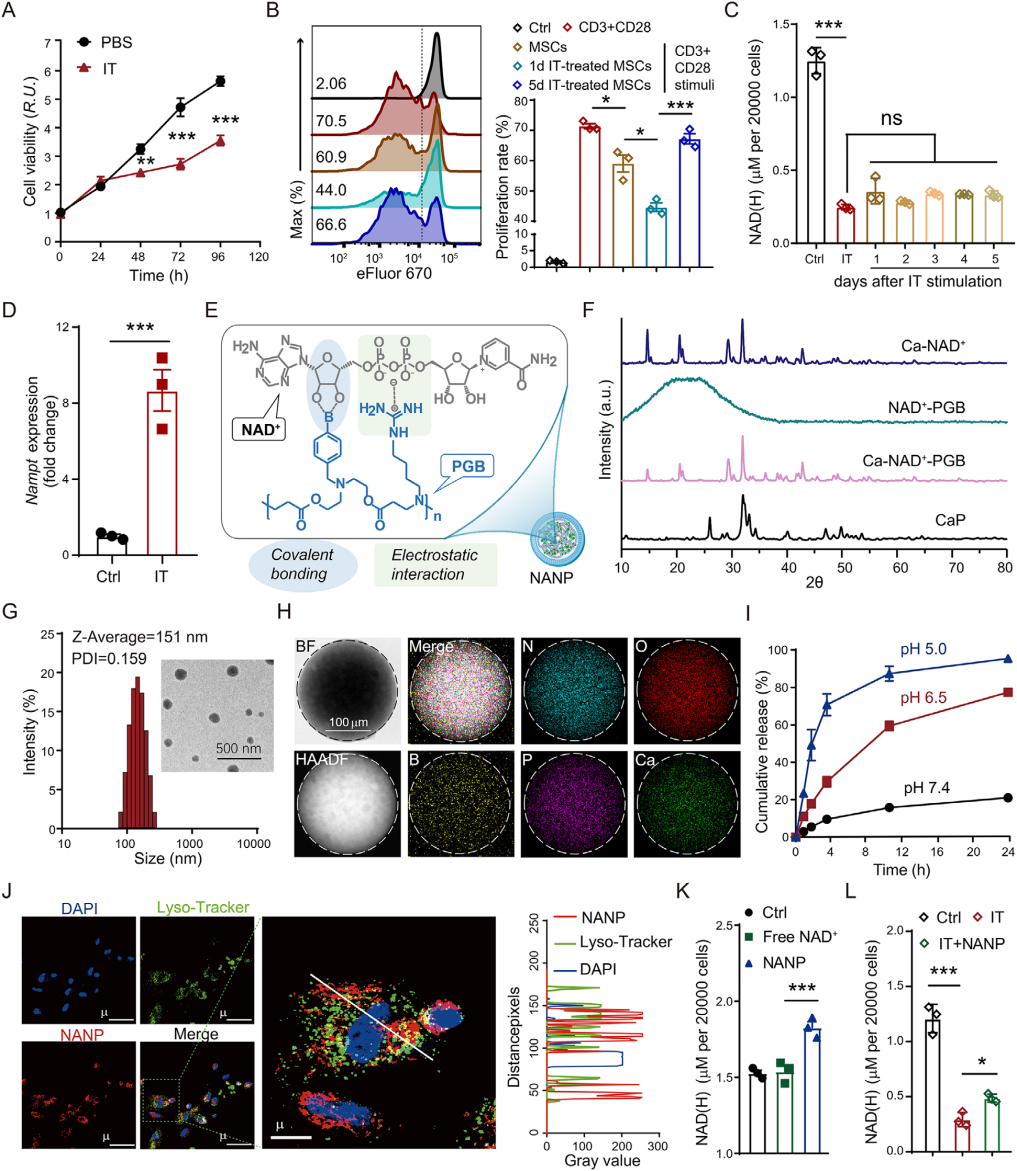
Preparation and characterization of NAD^+^ particles to maintain NAD^+^ levels in MSCs under inflammatory conditions. (A) MSCs viability with or without IT at the indicated time points was measured using the CCK‐8 assay, with statistical analyses presented in relative units (*R.U*.) (*n* = 3). (B) MSCs‐mediated suppression of anti‐CD3/CD28‐stimulated CD8^+^ T cell activation. “1d or 5d IT‐treated MSCs” indicates that MSCs were treated with IT for 1 day and 5 days, respectively. Representative flow cytometric dot plots and statistical analyses are shown (*n* = 3). (C) Intracellular NAD(H) levels in MSCs treated with IT for 24 h, along with daily NAD(H) measurements after IT stimulation (*n* = 3). (D) mRNA expression of *Nampt* in MSCs following IT stimulation for 24 h, as measured by qRT‐PCR (*n* = 3). (E) Schematic illustration of the interaction between NAD^+^ and PGB. (F) Powder XRD profiles of Ca‐NAD^+^, NAD^+^‐PGB, Ca‐NAD^+^‐PGB, and calcium phosphate (CaP). (G) Size and morphology of NANP characterized by dynamic light scattering and transmission electron microscopy. (H) The morphology characterization, HAADF image, and elemental mapping of Ca‐NAD^+^‐PGB nanoparticle. (I) The NAD(H) release profiles from NANP under different pH values. (J) Confocal imaging showing the co‐localization of NANP (red, labeled with DiD), lysosomes (green, stained by LysoTracker), and nuclei (blue, stained with Hoechst). Representative quantification of the mean fluorescence intensity (MFI) of NANP and LysoTracker is shown. (K) Intracellular NAD(H) levels of MSCs incubated with 25 µM free NAD^+^ or 25 µM NANP for 2 h (*n* = 3). (L) Intracellular NAD(H) levels of MSCs induced by IT with or without 25 µM NANP for 24 h (*n* = 3). All data were shown as mean ± SEM. *p < 0.05, **p < 0.01, ***p < 0.001; ns, not significant.

To further assess the immunoregulatory dynamics of MSCs under inflammatory stress, we evaluated their ability to suppress anti‐CD3/CD28‐stimulated CD8^+^ T cell proliferation following different durations of IT exposure. MSCs primed with IT for 1 day demonstrated increased suppression of CD8^+^ T cell proliferation. However, this effect was markedly diminished after five consecutive days of stimulation (Figure 1B), suggesting that sustained inflammatory signaling induces a transient anti‐inflammatory response followed by functional impairment of MSCs. This loss of function appears to be associated with a marked and sustained depletion of the intracellular NAD(H) pool, which persisted even after withdrawal of the inflammatory stimuli (Figure 1C). Although the key enzyme *Nampt* in NAD^+^ biosynthesis was upregulated (Figure 1D), it failed to restore intracellular NAD^+^ levels effectively. To directly test if NAD^+^ deficiency drives MSCs dysfunction, we inhibited its biosynthesis using FK866. This pretreatment impaired MSCs‐mediated CD8^+^T cell suppression, mirroring the effects of prolonged (5 days) IT stimulation (Figure S1H). These findings establish NAD^+^ depletion as a direct causal factor in MSCs dysfunction, connecting chronic inflammation to metabolic exhaustion.

It is inferred that NAD^+^ levels are critical for maintaining the long‐term therapeutic function of MSCs. Therefore, we next aimed to restore NAD^+^ within MSCs in the inflammatory microenvironment. A major challenge in replenishing NAD^+^ within cells lies in its nature as a hydrophilic nucleotide, which prevents it from crossing the cell membrane and being utilized effectively [21]. Alternative approaches, such as supplementing precursors like nicotinamide riboside (NR) or nicotinamide (NAM), are inefficient, and the synthesis of NAD^+^ is energy intensive. This issue is further aggravated during inflammation, where cells experience significant energy impairment [22].

To address this limitation, we developed an NAD^+^‐loaded nanoparticle (NANP) for efficient intracellular delivery of this small molecular nucleotide. Central to this system is a PBAE, rationally engineered with alternating phenylboronic acid and guanidinium groups that form strong multivalent interactions with the ribose‐phosphate backbone of NAD^+^ (Figure 1E). The x‐ray diffraction (XRD) analysis showed that NAD^+^ incorporated into the polymer exhibited a predominantly amorphous structure distinct from its crystalline form (Figure 1F). The polymer‐NAD^+^ complexes were then assembled into nanoparticles through a water‐in‐oil reverse microemulsion method [21], yielding uniform particles with an average diameter of 151 nm (Figure 1G). Elemental mapping further confirmed the co‐existence and homogeneous distribution of B, O, N, Ca, and P elements within the nanoparticles, illustrating the spatial integration of NAD^+^ with the engineered polymer network (Figure 1H).

This approach enabled an exceptionally high NAD^+^ loading content of 58.2%, significantly surpassing conventional carriers. The release of NAD^+^ from these nanoparticles is pH responsive, a property derived from the PGB polymer carrier. With a pKa of 6.32, PGB can sense subtle physiological pH variations. Its binding to nucleotides is strong at neutral pH but substantially weakened in acidic environments. Consequently, the nanoparticles efficiently release NAD^+^ under lysosomal acidic conditions, enabling controlled cytosolic delivery and sustained intracellular replenishment (Figure 1I). Confocal laser scanning microscopy (CLSM) was then applied to track the entry of NANP into MSCs. Within 4 h of transfection, NANP (indicated by red fluorescence) co‐localized with lysosomes (indicated by green fluorescence), confirming successful internalization of the nanoparticles into MSCs (Figure 1J). Notably, a significant amount of red fluorescence was observed outside the lysosomes, suggesting that NAD^+^ had successfully escaped from the lysosomes and was released into the cytosol. In comparison to free NAD^+^, NANP was more effective at replenishing the NAD(H) pool in MSCs (Figure 1K). This effect was particularly pronounced when MSCs were exposed to IT‐induced NAD^+^ depletion, with NANP increasing cellular NAD(H) levels by approximately 70% (Figure 1L). The above results indicate that the NAD^+^ nanoparticles we prepared can effectively restore intracellular NAD^+^ levels especially under inflammatory conditions.

### 2.2 NANP Enhance Self‐Persistence of MSCs to Improve Its Therapeutic Efficacy

We further evaluated the cytoprotective efficacy of NANP against IT‐induced damage. NANP supplementation completely reversed IT‐mediated growth arrest in MSCs, restoring proliferative capacity to baseline levels (Figure 2A). Inflammatory factors disrupt redox homeostasis, leading to excessive ROS generation, which in turn activates the mitochondrial permeability transition pore, causes loss of mitochondrial membrane potential, and facilitates the release of cytochrome C ultimately promoting apoptosis or necrosis [18, 23]. Consistent with this, we observed significant ROS generation and a loss of mitochondrial membrane potential following IT exposure (Figure 2B, Figure S2A), accompanied by an increase in cell death (Figure 2C). NANP treatment restored both mitochondrial membrane potential and viability. This protection may be attributed to the central role of NAD^+^ as an essential cofactor in energy metabolism and redox homeostasis.

**FIGURE 2:**
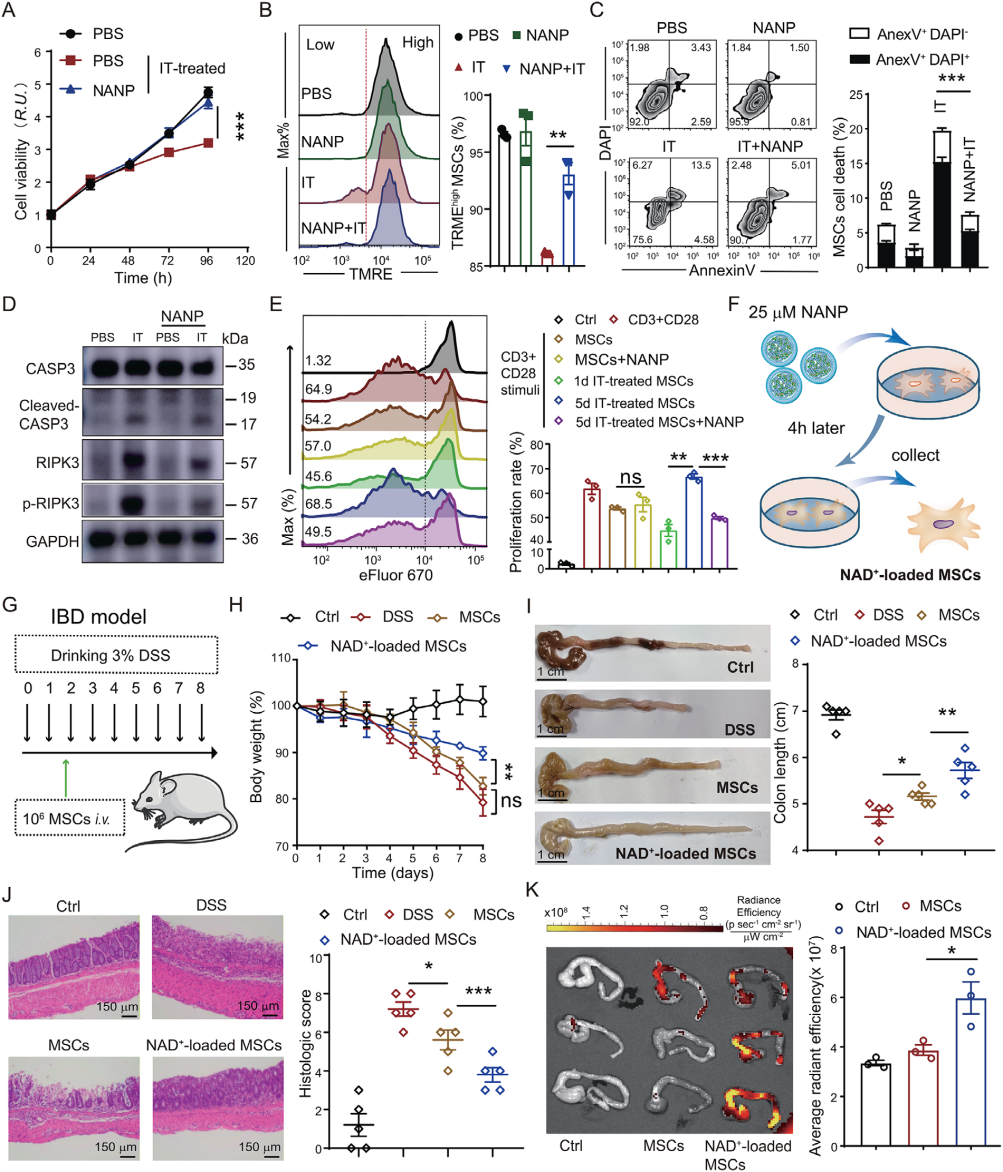
NAD^+^ supplementation protects MSCs from death and boosts their anti‐inflammatory potential in vivo. (A) The reduction in cell viability of MSCs after IT treatment can be reversed by the addition of 25 µM NANP (*n* = 3). (B) TMRE staining of MSCs treated with 25 µM NANP, IT, or their combination for 48 h, presented as cytofluorometric dot plots and statistical analysis (*n* = 3). (C) Annexin V/DAPI dual staining of MSCs treated with 25 µM NANP, IT, or their combination for 48 h, shown as cytofluorometric dot plots and statistical analysis (*n* = 3). (D) MSCs treated with 25 µM NANP, IT, or their combination for 48 h, the expression of apoptosis executors (CASP3 and cleaved CASP3), and necroptosis executors (RIPK3 and p‐RIPK3) was measured by semiquantitative WB. (E) 25 µM NANP restores the ability of 5‐days IT‐treated MSCs to inhibit anti‐CD3/CD28‐stimulated CD8^+^ T cell proliferation. Representative flow cytometric dot plots and statistical analyses are shown (*n* = 3). (F) Schematic diagram showing the preparation of NAD^+^‐loaded MSCs. (G) Schematic diagram showing the MSCs‐treated IBD model. (H) Body weight measurements of IBD mice were administered either MSCs or NAD^+^‐loaded MSCs (*n* = 5). (I) Colon length of IBD mice and quantitative analysis compared between DSS, MSCs, and NAD^+^‐loaded MSCs (*n* = 5). (J) Representative H&E‐stained colon sections and histological scores of IBD mice administered by MSCs or NAD^+^‐loaded MSCs (*n* = 5). (K) IVIS imaging of colon tissues. The mice were euthanized at 48 h after administration, and the colon was immediately removed for imaging with an IVIS Lumina Series (left panel). Average radiant efficiency was compared between MSCs and NAD^+^‐loaded MSCs (right panel) (*n* = 3). All data were shown as mean ± SEM. *p < 0.05, **p < 0.01, ***p < 0.001; ns, not significant.

Interestingly, despite pronounced oxidative stress and mitochondrial injury, IT exposure did not trigger caspase‐3‐mediated apoptosis. Instead, cell death occurred via RIPK3‐dependent necroptosis, as evidenced by the increased phosphorylation of RIPK3 and its downstream substrate MLKL, a hallmark of necroptotic cell death [24, 25] (Figure 2D, Figure S2C). NANP treatment markedly suppressed this inflammatory death pathway, suggesting that NAD^+^ restoration may interrupt necroptosis, potentially via the NLRC5 signaling axis [24]. Furthermore, NANP reduced IT‐induced expression of HLA‐DR (Figure S2B), a marker associated with MSCs immunogenicity, potentially lowering the risk of immune‐mediated clearance [26]. Notably, NANP restored the immunosuppressive function of MSCs after 5 days of IT exposure, allowing effective suppression of CD8^+^ T cell proliferation (Figure 2E). These results underscore the ability of NANP to preserve both the viability and functional integrity of MSCs under inflammatory stress.

Building on the findings above, we hypothesized that NAD^+^ supplementation would enhance the therapeutic efficacy of MSCs in inflammatory diseases. To test this, we pretreated MSCs with 25 µM NANP for 4 h to generate NAD^+^‐loaded MSCs (Figure 2F), and evaluated their therapeutic performance in a dextran sulfate sodium (DSS)‐induced murine colitis model (Figure 2G), a well‐established model of IBD. Compared to unmodified MSCs, free NAD^+^‐treated MSCs or empty nanoparticles (Empty NPs), a single intravenous injection of NAD^+^‐loaded MSCs markedly improved clinical outcomes, as demonstrated by faster weight recovery (Figure 2H, Figure S2D), less colon shortening (Figure 2I, Figure S2E,F), reduced disease activity index (DAI; Figure S2G,H), and alleviated histopathological features, including diminished bowel wall thickening and crypt loss (Figure 2J). Systemic inflammation was also ameliorated, as indicated by decreased serum IL‐6 levels, a key biomarker of colitis, in the NAD^+^‐loaded MSC group (Figure S2I). Moreover, colonic expression of E‐cadherin, a critical marker of epithelial integrity, was markedly elevated in colon tissues following NAD^+^‐loaded MSCs treatment (Figure S2J), suggesting enhanced epithelial repair and restoration of mucosal barrier function.

To further investigate the therapeutic advantage, we assessed the in vivo biodistribution of DiR‐labeled MSCs 48 h post‐injection. The cells were primarily distributed in the lungs, liver, and spleen (Figure S2K). Notably, MSCs preconditioned with NANP exhibited stronger fluorescence signals in the colon compared to control cells (Figure 2K), suggesting enhanced homing and persistence at the sites of inflammation. This improved tissue residency likely contributes to the superior therapeutic efficacy observed with NAD^+^‐replenished MSCs. Furthermore, serum levels of alanine aminotransferase (ALT) and aspartate aminotransferase (AST) remained within normal ranges across all experimental groups, indicating that neither MSCs administration nor NAD^+^‐loaded MSCs caused significant hepatic toxicity or other systemic side effects (Figure S2L).

### 2.3 NANP‐Preconditioned MSCs Modulate Colitic Immunity Through Suppression of Inflammatory Cell Trafficking

To assess how NAD^+^‐loaded MSCs reshape the intestinal immune landscape in IBD, we profiled leukocyte populations in colonic tissues, as immune cell infiltration is closely associated with mucosal injury and disease severity [27, 28]. Compared with untreated and control MSCs‐treated mice, NAD^+^‐loaded MSCs significantly reduced total leukocyte infiltration (Figure 3A, Figure S3A,B), indicating an overall attenuation of intestinal inflammation. Dendritic cells and CD4^+^ T cells, which are known to promote antigen presentation and sustain chronic inflammation in IBD [27], were notably decreased following NAD^+^‐loaded MSCs treatment (Figure 3B). Neutrophil levels also declined upon MSCs administration, although no significant difference was observed between unmodified and NAD^+^‐loaded MSCs (Figure 3B). In contrast, macrophages were increased in both proportion and absolute number in the NAD^+^‐loaded MSCs group (Figure 3C, Figure S3C). These cells exhibited elevated CD206 expression, as confirmed by flow cytometry and immunofluorescence staining (Figure 3C,D, Figure S3D,E), indicative of an anti‐inflammatory M2‐like phenotype. M2 macrophages are known to support inflammation resolution and epithelial repair. Collectively, these results demonstrate that NAD^+^ supplementation enhances the immunoregulatory impact of MSCs by suppressing pro‐inflammatory leukocytes and expanding reparative M2 macrophage populations in inflamed colonic tissues.

**FIGURE 3:**
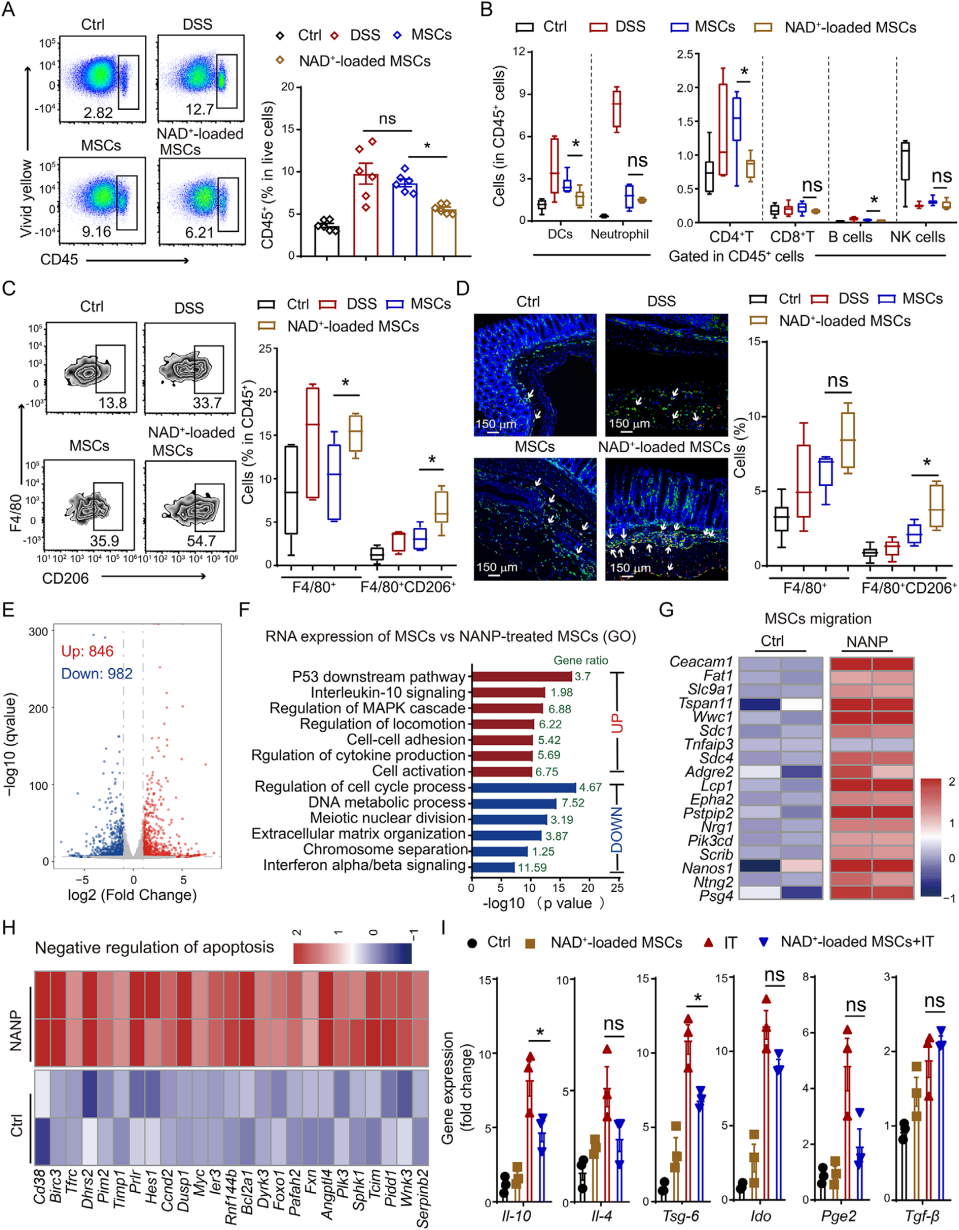
NANP‐primed MSCs reprogram colitic immunity by restraining inflammatory trafficking. (A) Proportion of CD45^+^ immune cells in the colon tissues of IBD mice, 6 days post‐MSCs therapy (*n* = 6). (B) Distribution of major colonic immune cell subsets, quantified by flow cytometry (*n* = 6). (C) Gating strategy for identifying F4/80^+^CD206^+^ macrophages, and statistical analyses are shown (*n* = 6). (D) Immunofluorescence imaging of colon sections depicting F4/80^+^CD206^+^ cells (green: F4/80, red: CD206, and blue: DAPI) in MSCs‐treated IBD mice, and statistical analyses were shown (*n* = 6). (E, F) RNA sequencing analysis identified differential gene expression in MSCs treated with PBS versus NANP, presented as a volcano plot (E) and Gene Ontology enrichment analysis (F) of significantly regulated genes. (G) Heatmap displaying the differential expression of genes involved in regulating cell migration, comparing PBS and NANP treatment. (H) Heatmap view of differential expression gene involved in negative regulation of apoptosis between MSCs and NANP‐treated MSCs. (I) The combination of 25 µM NANP with IT did not enhance anti‐inflammatory cytokine expression compared to IT alone in MSCs (*n* = 3). Data were shown as mean ± SEM. *p < 0.05, **p < 0.01, ***p < 0.001; ns, not significant.

To elucidate the molecular basis for NANP‐induced enhancement of MSCs function, we performed transcriptomic profiling of MSCs with or without NANP treatment. We identified 1828 differentially expressed genes (DEGs), with 846 genes upregulated and 982 genes downregulated (Figure 3E). Gene Ontology (GO) enrichment analysis revealed significant upregulation of genes associated with the p53 signaling pathway (Figure 3F), which regulates cell apoptosis and stress responses. Meanwhile, genes involved in migration and anti‐apoptosis were also upregulated (Figure 3G,H). These findings are consistent with previous studies showing that increased NAD^+^ availability activates the sirtuin family, particularly SIRT1, which functions as an NAD^+^‐dependent deacetylase to regulate transcriptional programs related to cellular senescence, stress resistance, genomic stability, and support cytoskeletal reorganization during migration [14, 16, 29, 30].

Although sequencing results demonstrated that NANP promotes the survival of MSCs under inflammatory conditions, they did not reveal that NANP enhanced the immune regulatory function of MSCs themselves. It is well established that MSCs exert their immunomodulatory effects primarily through a robust secretome, which includes a variety of cytokines [31]. Based on our earlier results showing that NAD^+^ supplementation enhances the immunosuppressive function of MSCs, we initially hypothesized that this improvement might be due to increased production of these soluble mediators. Therefore, we supplemented MSCs with NANP, and surprisingly found that this supplementation did not significantly increase the expression of immunomodulatory factors including IL‐10, IL‐4, IDO, PGE2, and TGF‐β (Figure 3I), which were typically upregulated in response to inflammatory stimuli [31, 32]. Additionally, the combination of MSCs and IT resulted in a mild decrease in these factors compared to IT alone, particularly IL‐10 and TSG‐6. These observations suggest that the enhanced immunomodulatory activity of NAD^+^‐loaded MSCs is not mediated by increased cytokine secretion but likely involves alternative mechanisms.

### 2.4 NANP Reshapes the Immune Modulatory Properties of MSCs‐Derived Exosomes

MSCs‐derived exosomes have long been recognized for their anti‐inflammatory properties. These nanoscale vesicles, enriched with regulatory microRNAs, proteins, and lipids, are capable of orchestrating immune responses through multiple mechanisms. They promote macrophage polarization from the pro‐inflammatory M1 to the anti‐inflammatory M2 phenotype, suppress Th1 and Th17 responses while favoring Th2 and Treg expansion, and attenuate dendritic cells activity by impairing antigen uptake and inhibiting maturation. Such attributes render MSCs‐exo critical effectors of immunomodulation in inflammatory diseases [33].

In our study, these immunosuppressive capabilities were reflected in the immune landscape of colitic mice treated with NAD^+^‐loaded MSCs. To investigate whether the therapeutic effect of NANP is exosome dependent, we administered GW4869, a specific inhibitor of exosome biogenesis. This intervention significantly reversed the therapeutic benefits conferred by NAD^+^‐loaded MSCs, as indicated by impaired recovery of body weight (Figure S4A), reduced preservation of colon length (Figure S4B), and worsened DAI scores (Figure S4C). To further explore the immunomodulatory role of exosomes, we isolated exosomes from MSCs, NAD^+^‐loaded MSCs, IT‐treated MSCs, and IT + NAD^+^‐loaded MSCs (Figure S4D). Using an ex vivo antigen presentation system with OVA‐pulsed bone marrow‐derived dendritic cells (BMDC), we examined the ability of these exosomes to modulate OT‐I CD8^+^ T cell proliferation (Figure 4A). Exosomes from MSCs exhibited a baseline ability to suppress T cell proliferation, which was markedly enhanced following NAD^+^ loading. While inflammatory (IT) stimulation slightly impaired this suppressive effect, concurrent NAD^+^ supplementation not only reversed the decline but significantly boosted the immunosuppressive potency of MSCs‐exo, regardless of IT exposure (Figure 4B). This divergence highlights a key finding that NAD^+^ supplementation enhances the immunosuppressive capacity of MSCs‐exo in both steady‐state and inflammatory conditions, countering functional decline induced by sustained inflammatory stress.

**FIGURE 4:**
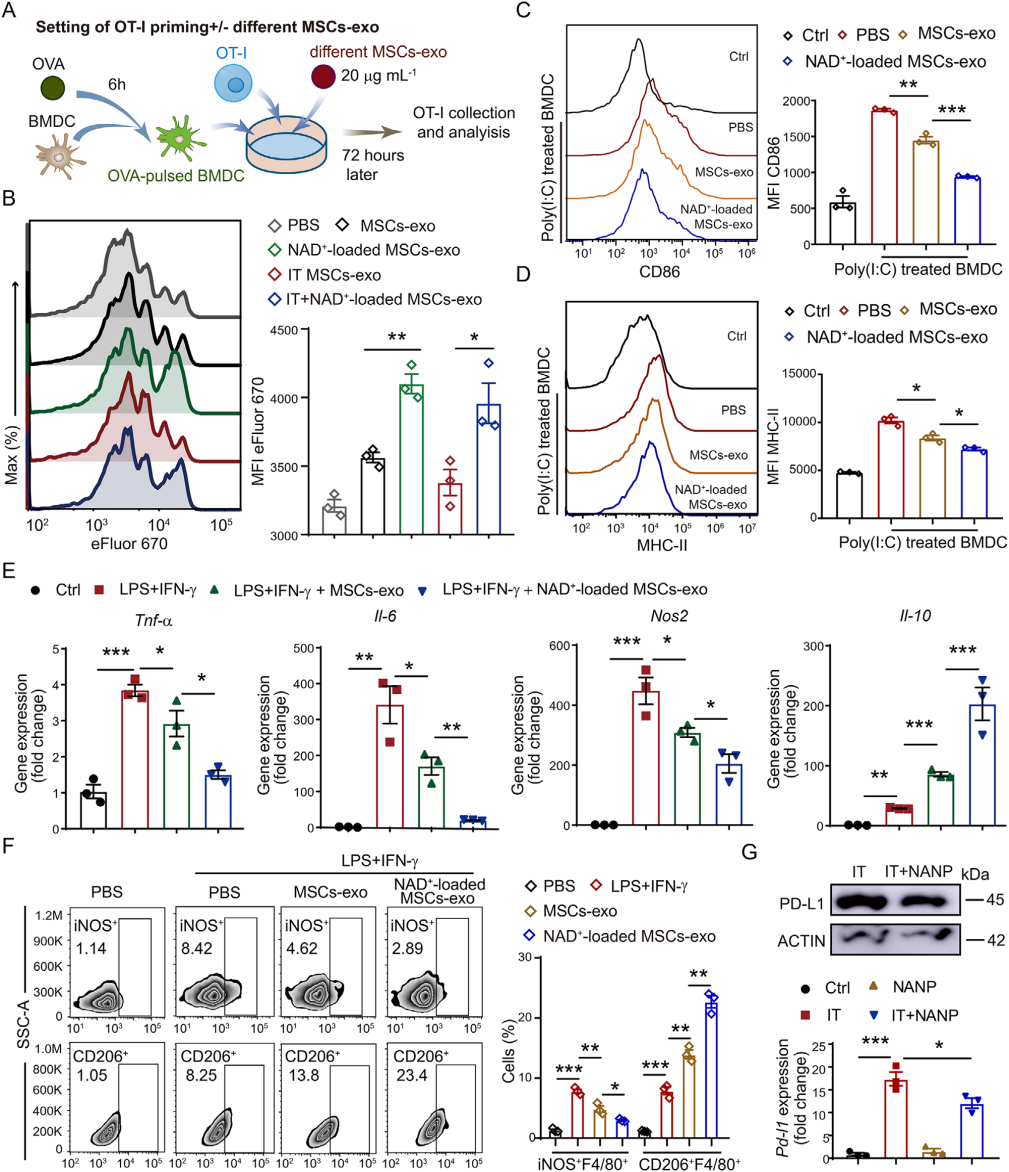
NANP alters the immunomodulatory properties of MSCs‐exo. (A) Strategy for in vitro MSCs‐exo‐mediated inhibition of OT‐I cell proliferation. (B) Representative histograms showing the proliferation of eFluor670‐labeled OT‐I cells in vitro. Statistical analysis of OT‐I cell‐associated MFI is shown (*n* = 3). (C, D) Representative histograms illustrating CD86 (C) and MHC‐II (D) expression in BMDC after Poly(I:C) stimulation with or without different MSCs‐exo. Statistical analysis of mean fluorescence intensity (MFI) is shown (*n* = 3). (E, F) RAW264.7 cells were pretreated with LPS (100 ng mL^−1^) and IFN‐γ (10 ng mL^−1^) for 2 h, and then cultured with 20 µg mL^−1^ exosomes from MSCs or NAD^+^‐loaded MSCs for another 12 h. The mRNA expressions of *Tnf‐a, Il‐6, Nos2*, and *Il‐10* genes were detected by using qRT‐PCR assay (E) (*n* = 3), and the proportions of iNOS^+^F4/80^+^ and CD206^+^F4/80^+^ cells were detected by flow cytometry (F) (n = 3). (G) Relative *Pd‐l1* mRNA levels in MSCs treated with IT, 25 µM NANP, or a combination of both for 24 h (bottom panel), and Western blot analysis of PD‐L1 in exosomes derived from MSCs treated with 25 µM NANP or not (top panel). Data were shown as mean ± SEM. *p < 0.05, **p < 0.01, ***p < 0.001; ns, not significant.

We then turned to the biogenesis and function of exosomes under NAD^+^‐replenished conditions. Transmission electron microscopy (TEM) and dynamic light scattering (DLS) analysis confirmed their characteristic morphology and size distribution (50–150 nm), along with the presence of classical exosomal markers such as CD63, CD81, TSG101, and ALIX (Figure S4E,F). Notably, NANP treatment enhanced exosome secretion by over 1.5‐fold, as indicated by the nanoparticle tracking analysis (NTA) quantification (Figure S4G). Fluorescence imaging and FACS analysis using DiO labeling confirmed that these MSCs‐exo were effectively internalized by both RAW264.7 and BMDC (Figure S4H,I). Furthermore, exosomes from NANP‐treated MSCs significantly inhibited Poly(I:C)‐induced BMDC maturation, as evidenced by downregulation of CD86 and MHC class II expression (Figure 4C,D) [34]. Additionally, compared to free NAD^+^ and Empty NPs, NANP enhanced the ability of MSCs‐exo to promote M2 macrophage polarization in vitro, as shown by decreased expression of pro‐inflammatory genes (*Tnf‐α*, *Il‐6*, and *Nos2*) and increased expression of the anti‐inflammatory cytokine *Il‐10* (Figure 4E, Figure S4J), alongside diminished iNOS and elevated CD206 expression (Figure 4F). These findings are consistent with our in vivo results, confirming that NAD^+^‐loaded MSCs foster immune tolerance in IBD primarily through exosome‐mediated modulation of the inflammatory microenvironment.

We next investigated how NANP modulates the immunoregulatory function of MSCs‐exo. Given the reported role of PD‐L1 in mediating exosomes‐based immunosuppression [35, 36], we speculated that NAD^+^ supplementation might enhance this effect via PD‐L1 upregulation. To test this, we measured PD‐L1 expression in both MSCs and their exosomes following NANP treatment. Although IT stimulation significantly increased *Pd‐l1* mRNA levels in MSCs, NANP treatment surprisingly did not further increase *Pd‐l1* expression (Figure 4G). Moreover, PD‐L1 protein levels in MSCs‐exo remained unchanged following NAD^+^ loading. These results suggest that PD‐L1 is not the primary driver of the enhanced immunomodulatory activity conferred by NANP.

### 2.5 NANP Modulates miRNA Content in Exosomes via SMPD3 Upregulation

Exosomes efficiently deliver molecular signals, such as proteins, lipids, DNA, and RNA, between cells. Among these, miRNAs are of particular interest due to their central role in gene silencing and immune modulation in recipient cells [37, 38]. Once delivered, exosomal miRNAs can reshape target gene expression, revealing a previously underappreciated form of intercellular communication. Given this, we hypothesized that specific miRNAs enriched in NAD^+^‐loaded MSCs‐derived exosomes might account for their enhanced immunoregulatory activity. To prove this, we analyzed miRNA profiles in MSCs‐exo by small RNA deep sequencing, and found that NAD^+^‐loaded MSCs‐exo upregulated 26 miRNAs and downregulated 48 miRNAs compared to MSCs‐exo (Figure 5A). Gene Ontology (GO) enrichment analysis further revealed that these differentially expressed miRNAs were predominantly involved in immune regulation, with a particular focus on pathways related to negative regulation of TLR signaling and type I interferon responses—both critical for macrophage and dendritic cell activation (Figure 5B) [39, 40].

**FIGURE 5:**
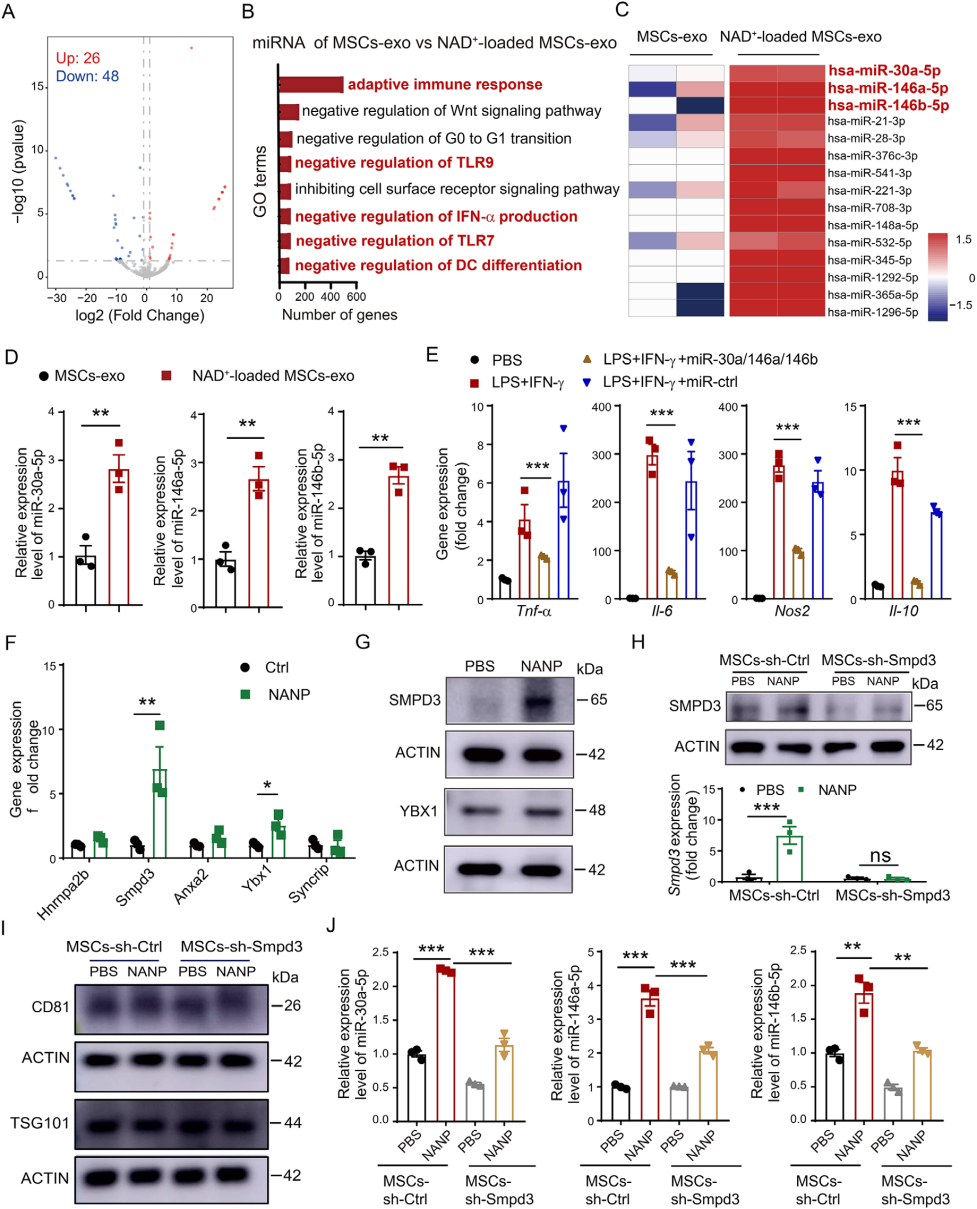
NANP modulates the miRNA content in exosomes through the upregulation of SMPD3. (A) Volcano plot showing the differential expression analysis of miRNAs between MSCs‐exo and NAD^+^‐loaded MSCs‐exo. (B) GO analysis of significantly enriched genes in NAD^+^‐loaded MSCs‐exo compared to MSCs‐exo. (C) Heatmap illustrating the upregulation of miRNAs in NAD^+^‐loaded MSCs‐exo compared to MSCs‐exo. (D) qRT‐PCR analysis of miR‐30a‐5p, miR‐146a‐5p, and miR‐146b‐5p expression in exosomes from MSCs treated with or without NANP. (E) RAW264.7 cells were pretreated with LPS (100 ng mL^−1^) and IFN‐γ (10 ng mL^−1^) for 2 h, followed by transfection with siRNA‐ctrl or a combination of miR‐30a‐5p, miR‐146a‐5p, and miR‐146b‐5p mimics (miR‐30a/146a/146b) for an additional 24 h. The mRNA expressions of the *Tnf‐a*, *Il‐6*, *Nos2*, and *Il‐10* genes were detected using qRT‐PCR (*n* = 3). (F) The mRNA expressions of *Hnrnpa2b1*, *Smpd3*, *Anxa2*, *Ybx1*, and *Syncrip* of MSCs with or without 25 µM NANP treatment for 48 h (*n* = 3). (G) Protein levels of SMPD3 and YBX1 in MSCs with or without NANP (25 µM, 48 h) treatment. (H) Western blot and qRT‐PCR showing that MSCs with shRNA‐mediated SMPD3 knockdown (MSCs‐sh‐Smpd3) fail to respond to NANP‐induced SMPD3 upregulation. (I) Western blot analysis of the exosomal markers TSG101 and CD81 detected in exosomes derived from MSCs‐sh‐Ctrl and MSCs‐sh‐Smpd3 cells. (J) The expression of miR‐30a‐5p, miR‐146a‐5p, and miR‐146b‐5p in exosomes derived from MSCs‐sh‐Ctrl and MSCs‐sh‐Smpd3 in the presence or absence of NANP, measured by qRT‐PCR (*n* = 3). Data were shown as mean ± SEM. *p < 0.05, **p < 0.01, ***p < 0.001; ns, not significant.

Among the upregulated miRNAs, three highly differentially expressed miRNAs (miR‐30a‐5p, miR‐146a‐5p, and miR‐146b‐5p) were highlighted for their anti‐inflammatory properties (Figure 5C) [41, 42, 43], whereas the role of many downregulated miRNAs remains poorly understood (Figure S5A). qRT‑PCR confirmed that NANP preconditioning upregulated the expression of these three specific miRNAs in MSCs‐exo (Figure 5D). MSCs‐exo were then co‑cultured with RAW264.7 macrophages and BMDC, and the corresponding miRNAs were detected in the recipient cells, with notably higher levels observed following treatment with exosomes from NANP‑preconditioned MSCs (Figure S5B). To evaluate the functional contribution of these miRNAs, RAW264.7 macrophages were transfected with a miR‐30a/146a/146b combination. As expected, this cocktail significantly suppressed the LPS plus IFN‑γ‑induced expression of pro‑inflammatory genes (*Tnf‑α*, *Il‑6*, and *Nos2*) (Figure 5E). Interestingly, *Il‑10* expression was also reduced, which may reflect a broader inhibition of TLR signaling, consistent with previous reports [44]. Moreover, transfection of inhibitors targeting these miRNAs into recipient cells markedly reversed the observed anti‑inflammatory effects, confirming that these exosomal miRNAs are essential for mediating the immunoregulatory outcome (Figure S5C,D). Together, these findings indicate that the enhanced anti‑inflammatory activity of NAD^+^‑loaded MSCs‑exo is largely attributable to the upregulation of these three miRNAs.

Previous studies have shown that cells regulate miRNA packaging in exosomes through distinct sorting mechanisms [45, 46]. To explore whether NAD^+^ supplementation via NANP influences these mechanisms in MSCs‐exo, particularly in relation to the enrichment of miR‐30a/146a/146b, we examined the expression of five miRNA sorting‐related genes. Among them, NANP treatment markedly elevated both mRNA and protein levels of SMPD3 (nSMase2), a key enzyme involved in ceramide‐mediated exosome biogenesis. The mRNA levels of YBX1 also increased, but to a lesser extent than SMPD3 (Figure 5F,G). Prior work has established SMPD3 as a central regulator in both RNA‐binding protein‐dependent and ceramide‐dependent sorting pathways, facilitating miRNA enrichment into extracellular vesicles [46, 47]. However, whether SMPD3 specifically governs the sorting of miR‐30a/146a/146b has not been previously reported.

To address this, we knocked down SMPD3 in MSCs using shRNA. Western blotting and qRT‐PCR confirmed efficient knockdown in MSCs‐sh‐Smpd3 cells relative to MSCs‐sh‐Ctrl controls (Figure 5H). We isolated exosomes from both groups and confirmed the expression of typical markers such as CD81 and TSG101 (Figure 5I). Notably, SMPD3 deficiency significantly reduced total exosome production (Figure S5E), confirming its role in exosome biogenesis. Furthermore, the ability of NANP to enrich miR‐30a‐5p, miR‐146a‐5p, and miR‐146b‐5p in exosomes was significantly compromised in MSCs with SMPD3 knockdown, as their levels were markedly lower than in exosomes from SMPD3‐intact MSCs. This indicates that the depletion of SMPD3 disrupts the ability of NANP to enhance the selective loading of these specific miRNAs into exosomes. Importantly, despite NANP treatment, exosomes from SMPD3‐deficient MSCs failed to suppress LPS and IFN‐γ‐induced pro‐inflammatory cytokine expression (*Tnf‐α*, *Il‐6*, and *Nos2*) in RAW264.7 macrophages (Figure S6A). These exosomes also failed to inhibit Poly(I:C)‐induced dendritic cells maturation (Figure S6B). Collectively, these findings identify SMPD3 as a key mediator of NANP‐enhanced miRNA sorting into exosomes and establish its essential role in the exosome‐driven immunomodulatory functions of NAD^+^‐replenished MSCs.

### 2.6 NAD^+^/α‐KG‐Mediated TET1 Demethylation Regulates SMPD3 Expression

Building on the observation that SMPD3 knockdown compromises the immunomodulatory capacity of MSCs‐exo, we next examined whether this deficiency would undermine their therapeutic efficacy in the IBD model. Consistent with our hypothesis, SMPD3 deficiency abolished the therapeutic benefit of NANP reprogramming, as evidenced by greater body weight loss and elevated DAI (Figure 6A, Figure S6C). Additionally, treatment with NAD^+^‐loaded MSCs‐sh‐Smpd3 failed to alleviate colon shortening, and histological analysis revealed persistent bowel wall thickening and crypt damage. In contrast, these pathological features were markedly improved in mice treated with NAD^+^‐loaded MSCs‐sh‐Ctrl (Figure 6B,C). These findings indicate that SMPD3 expression is essential for the therapeutic efficacy of NANP‐pretreated MSCs. Flow cytometry analysis revealed that SMPD3 knockdown completely abolished the immunomodulatory effects of NAD^+^‐reprogrammed MSCs, as evidenced by the loss of F4/80^+^CD206^+^ macrophage enrichment and the failure to reduce dendritic cells infiltration in the colon tissue (Figure S6D).

**FIGURE 6:**
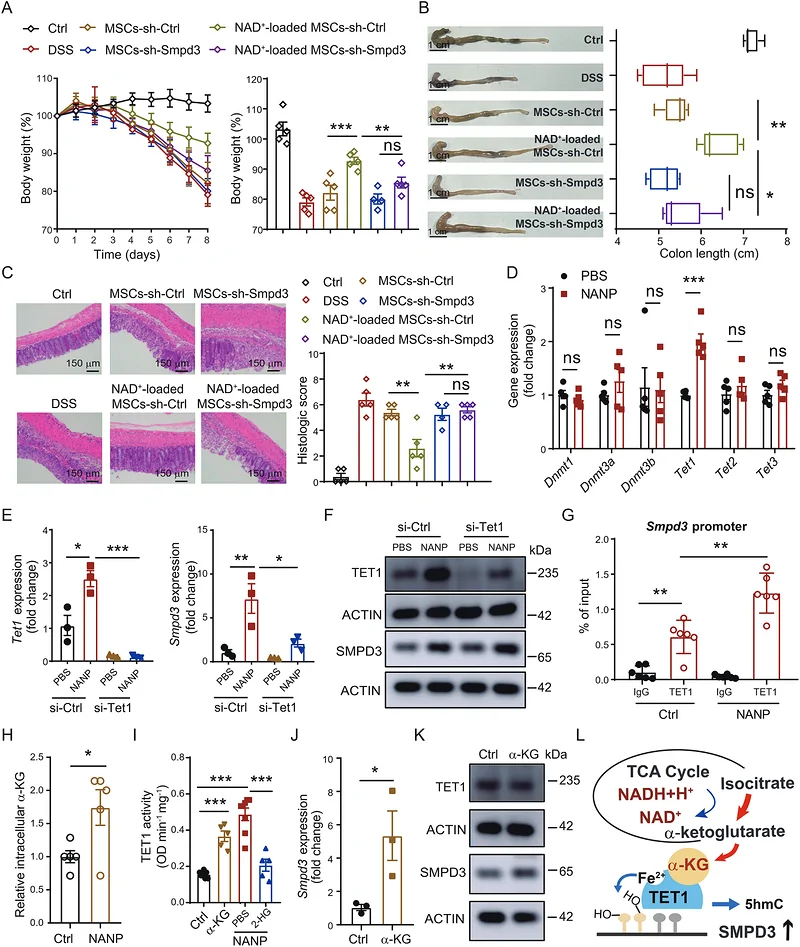
The NAD^+^/α‐KG‐TET1‐SMPD3 axis regulates the exosomal function of MSCs. (A) Body weight of IBD mice treated with MSCs‐sh‐Ctrl or MSCs‐sh‐Smpd3, with or without NANP pre‐treatment (25 µM, 4 h) (left panel), and the corresponding statistical analysis (right panel) (*n* = 4–5). (B) Colon length of IBD mice administered MSCs‐sh‐Ctrl or MSCs‐sh‐Smpd3, with or without NANP (25 µM, 4 h) pre‐treatment (left panel), and quantitative analysis (right panel) (*n* = 4–5). (C) Representative H&E‐stained colon sections and histological scores of IBD mice treated with MSCs‐sh‐Ctrl or MSCs‐sh‐Smpd3, with or without NANP treatment (25 µM, 4 h) (*n* = 4–5). (D) The mRNA levels of key regulatory enzymes for DNA methylation (DNMTs) and demethylation (TETs) were measured by qRT‐PCR in MSCs following treatment with or without 25 µM NANP for 24 h (*n* = 3). (E) The mRNA expression levels of *Tet1* and *Smpd3* in MSCs transfected with Tet1‐siRNA, in the presence or absence of 25 µM NANP for 24 h (*n* = 3). (F) Protein levels of TET1 and SMPD3 in MSCs transfected with Tet1‐siRNA, with or without 25 µM NANP for 24 h. (G) TET1 binding to the *Smpd3* promoter in MSCs, assessed by ChIP‐qPCR, IgG served as a control (*n* = 6). (H) Relative intracellular α‐KG levels in MSCs after treatment with 25 µM NANP for 24 h (*n* = 5). (I) TET1 enzymatic activity in cell lysates from MSCs treated with NANP, NANP plus 2‐HG, or α‐KG for 24 h (*n* = 5–8). (J) The mRNA expression levels of *Smpd3* from MSCs treated with 1 mM α‐KG for 24 h (*n* = 3). (K) Protein levels of TET1 and SMPD3 in MSCs with or without 1 mM α‐KG supplement for 24 h. (L) Schematic illustrating how NANP promotes α‐KG production, thereby regulating TET1‐mediated demethylation of SMPD3. Data were shown as mean ± SEM. *p < 0.05, **p < 0.01, ***p < 0.001; ns, not significant.

To explore how NAD^+^ regulates SMPD3 expression, we first considered the role of SIRT1, a well‐known NAD^+^‐dependent deacetylase [48]. SIRT1 has previously been linked to SMPD3 regulation during the progression from steatosis to MASH [49]. However, treatment with NAM, an NAD^+^ analog that inhibits SIRT1 activity by occupying its NAD^+^ ‐binding pocket [50], did not reduce SMPD3 expression in MSCs (Figure S6E), indicating that NANP‐induced SMPD3 expression is independent of SIRT1‐mediated deacetylation.

DNA methylation is a fundamental epigenetic mechanism that controls gene expression by modulating chromatin structure and transcription factor accessibility. Promoter hypermethylation is frequently linked to transcriptional repression. Prior studies have demonstrated that hypermethylation of the SMPD3 promoter suppresses its expression across various cell types [51]. Based on this evidence, we investigated whether NAD^+^ supplementation could influence the methylation status of SMPD3 and thereby facilitate its transcriptional activation. Therefore, we examined the expression of key regulators involved in DNA methylation and demethylation, including DNA methyltransferases (DNMTs) that maintain or establish methylation patterns, and the ten‐eleven translocation (TET) dioxygenases, which catalyze active DNA demethylation through the oxidation of 5‐methylcytosine (5mC) [52]. Our analysis revealed that NANP treatment did not significantly alter the expression of DNMTs. In contrast, TET1 exhibited pronounced upregulation in response to NAD^+^ supplementation (Figure 6D). These findings suggest that NAD^+^ supplementation may enhance SMPD3 expression through TET1‐mediated promoter demethylation. To further validate the role of TET1 in promoting the demethylation of SMPD3, we performed TET1 knockdown experiments using siRNA transfection. The results demonstrated that silencing TET1 significantly attenuated the upregulation of both TET1 and SMPD3 induced by NANP (Figure 6E,F), indicating that TET1 is essential for the NANP‐mediated activation of SMPD3 expression. These results provide initial evidence linking NAD^+^ metabolism to epigenetic remodeling pathways that control exosome biogenesis and function.

TET1 plays a pivotal role in epigenetic regulation by catalyzing the conversion of 5mC to 5‐hydroxymethylcytosine (5hmC), a key step in active DNA demethylation. Previous studies have shown that both TET1 and 5hmC tend to localize preferentially at the transcriptional start sites of CpG‐rich promoters [53]. Using Methprimer software [54], we examined a 2‐kb segment within the *Smpd3* promoter region and identified a CpG island (Figure S6F), prompting us to investigate whether *Smpd3* is a direct target of TET1. We then performed ChIP‐qPCR analysis, which confirmed that TET1 binds to the CpG island of the *Smpd3* promoter. Notably, NANP treatment enhanced this binding (Figure 6G), likely due to the increased expression of TET1 induced by NANP.

α‐KG is a critical co‐substrate for Fe^2+^‐ and 2‐oxoglutarate‐dependent dioxygenases, including the TET family of enzymes, which are essential for DNA demethylation [55]. Within the tricarboxylic acid (TCA) cycle, α‐KG is largely produced via the NAD^+^‐dependent oxidative decarboxylation of isocitrate, thereby directly linking NAD^+^ metabolism to TET enzymatic activity. Consistent with this metabolic–epigenetic coupling, NANP treatment significantly elevated intracellular α‐KG levels (Figure 6H). Exogenous α‐KG treatment markedly enhanced TET1 catalytic activity, an effect recapitulated by NANP supplementation (Figure 6I). Critically, this NANP‐induced activation of TET1 was completely abolished by 2‐hydroxyglutarate (2‐HG), a potent competitive inhibitor that blocks α‐KG binding, underscoring the essential role of α‐KG‐dependent catalysis in mediating the epigenetic effects of NAD^+^ supplementation.

In parallel, exogenous α‐KG supplementation also increased *Smpd3* expression without altering TET1 protein levels (Figure 6J,K). This is consistent with α‐KG enhancing TET1 enzyme activity, thereby promoting *Smpd3* demethylation and subsequent gene expression. However, as α‐KG does not increase TET1 abundance, it cannot recruit additional TET1 to the *Smpd3* locus (Figure S6G). Taken together, these results demonstrate that NAD^+^ supplementation elevates α‐KG production, which in turn potentiates TET1 enzymatic activity to drive SMPD3 promoter demethylation, thereby augmenting the immunomodulatory capacity of MSCs‐exo (Figure 6L).

## 3 Discussion

MSCs have gained considerable attention as immunomodulatory agents for the treatment of inflammatory diseases. Yet, their clinical translation remains hampered by inconsistent outcomes, largely due to their limited persistence and functional instability in hostile inflammatory milieus [56]. Despite sustained progress in the field, the majority of MSCs‐based interventions remain confined to early‐phase clinical evaluation (Phases I and II) [57], underscoring the enduring difficulty of preserving MSCs functionality under pathological stress. Recent work implicated NAD^+^ metabolism as a central regulator of cellular adaptation to inflammatory stress. Under acute inflammatory stress, MSCs activate the NAD^+^ salvage pathway to reinforce glycolysis and sustain immunosuppressive activity via the succinate‐HIF‐1α axis, highlighting NAD^+^ as a key driver of their immunoregulatory capacity [12]. Extending these insights, our study demonstrates that NAD^+^ replenishment not only preserves MSCs survival and functionality under inflammatory stress, but also reveals a metabolism–epigenetics–exosome axis essential for their paracrine immunomodulatory function.

In this study, we observed that sustained inflammatory exposure drives MSCs into an exhaustion‐like state marked by profound metabolic collapse and epigenetic instability. A hallmark feature of this process is the rapid and irreversible depletion of NAD^+^, exacerbated by the heightened glycolytic flux that accompanies cellular stress. Although NAMPT is transcriptionally upregulated in a compensatory attempt to restore NAD^+^, its enzymatic activity is compromised under inflammatory conditions due to direct inhibition and disrupted AMP/ATP balance [58]. The depletion of NAD^+^, as shown by our data, not only undermines cellular metabolism but also renders MSCs vulnerable to inflammatory cell death pathways. Indeed, collapse of the NAD^+^ pool triggers PANoptosis and is consistent with observations that MSCs under inflammatory stress exhibit heightened susceptibility to RIPK1‐driven necroptosis [24, 59]. Similarly, NAD^+^ depletion has been identified as a central pathogenic mechanism in inflammatory disorders such as colitis and nephritis [60, 61]. These observations demonstrate that reliance on the NAD^+^ salvage pathway alone is insufficient to sustain NAD^+^ homeostasis in MSCs under persistent inflammatory conditions, highlighting the indispensable role of exogenous NAD^+^ replenishment in preserving both MSCs survival and their immunoregulatory capacity.

Direct supplementation of NAD^+^ is inefficient because its hydrophilic, negatively charged nature prevents membrane penetration [21]. Precursor‐based strategies, such as NAM or NR, are also suboptimal, since conversion to NAD^+^ is energetically costly and particularly ineffective under inflammatory stress. To address this limitation, we designed a nanoparticle system that encapsulates NAD^+^ and enables controlled lysosomal release, achieving markedly enhanced intracellular delivery and providing a reliable means to replenish NAD^+^ even in hostile inflammatory conditions. Following NAD^+^ restoration, MSCs displayed heightened resistance to inflammation‐induced cell death, growth arrest, and the exhaustion‐like states, accompanied by a marked recovery of their immunoregulatory activity. Notably, whereas many current strategies to enhance MSCs function focus on fortifying intrinsic metabolic capacity or augmenting anti‐inflammatory mediator production, our findings revealed that NAD^+^ supplementation did not increase the secretion of canonical factors such as IL‐10, IDO, PGE2, or TSG‐6.

In contrast to intrinsic mediators, our work reveals that NAD^+^ supplementation exerts its most profound effect through the exosomal axis. NAD^+^ repletion not only increases the quantity of exosomes secreted by MSCs but also strengthens their immunoregulatory efficacy, markedly improving the suppression of dendritic cell maturation and the induction of macrophage M2 polarization. Importantly, after excluding contributions from surface immunomodulators such as PD‐L1, we identified a robust enrichment of several regulatory microRNAs‐including miR‐30a‐5p, miR‐146a‐5p, and miR‐146b‐5p‐in NAD^+^‐primed exosomes. These miRNAs converge on key inflammatory nodes, including TRAM, TLR/IL‐1R pathways, and IKKβ, thereby attenuating NF‐κB‐driven responses [62].

Further investigation revealed that SMPD3 plays a pivotal role in regulating both exosome biogenesis and the selective loading of immunoregulatory miRNAs [46]. SMPD3 catalyzes the hydrolysis of sphingomyelin to ceramide, thereby driving ceramide‐dependent inward budding of endosomal membranes and formation of multivesicular bodies [63]. Previous studies have shown that SMPD3 overexpression enhances exosome‐based delivery systems, enhances MSCs functionality, and even promotes bone regeneration under metabolic stress [64]. Conversely, pharmacological inhibition of SMPD3 alters miRNA release, highlighting its critical role in exosomal cargo sorting. Silicate stimulation in endothelial progenitor cells was reported to induce SMPD3 expression, leading to enrichment of exosomal miR‐126a‐3p and enhanced angiogenesis after myocardial infarction [45]. Our study demonstrates that SMPD3 upregulation in MSCs markedly enriches a panel of inflammation‐regulating miRNAs, showing that metabolic modulation can reprogram exosomal cargo at the level of miRNA selection. These results establish SMPD3 as a mechanistic link between intracellular metabolism and the paracrine immunoregulatory function of MSCs.

Our work also identified TET1, a Fe^2+^/α‐KG‐dependent methylcytosine dioxygenase that promotes DNA demethylation, as a pivotal upstream regulator of SMPD3. TET1 is well recognized for orchestrating gene expression programs relevant to stem cell differentiation and immune regulation [65]. Prior investigations have demonstrated that TET1 activation augments the transcription of immune mediators such as CXCL10 and CCL5, while simultaneously protecting against maladaptive metabolic reprogramming [66]. Conversely, loss of TET1 function reduces the expression of miR‐30b‐5p, thereby exacerbating neuronal hyperexcitability and inflammatory responses [67]. In our work, ChIP‐qPCR analysis confirmed that TET1 directly binds the CpG island within the Smpd3 promoter, establishing direct transcriptional control over its activity. Previous studies have provided strong evidence that intracellular NAD^+^ enhances TET1 enzymatic activity through α‐KG‐dependent mechanisms [68]. Extending this cascade, our study demonstrates that TET1 directly drives SMPD3 expression, thereby linking NAD^+^ availability to exosomal biogenesis and cargo selection.

While our results support the therapeutic benefits of NAD^+^‐elevated MSCs, potential long‐term safety and translational considerations warrant careful evaluation. In our design, NAD^+^ elevation is achieved through a short in vitro pretreatment rather than sustained in vivo supplementation. Given the rapid metabolic turnover of NAD^+^, intracellular levels are dynamically regulated rather than persistently accumulated. Our preliminary data indicate that NANP treatment does not adversely affect MSCs proliferation or alter their immunophenotype, suggesting preserved cellular identity and function. The PGB carrier is biodegradable, and transient exposure with subsequent washing steps reduces the likelihood of material persistence within MSCs or their secreted exosomes. Nevertheless, systematic long‐term safety evaluation and donor‐to‐donor validation will be necessary prior to clinical translation.

In summary, our study demonstrates that persistent inflammatory stress drives MSCs into an exhaustion‐like state through irreversible NAD^+^ depletion, resulting in metabolic collapse, cell death, and loss of immunoregulatory function. By directly restoring NAD^+^ using a rationally designed nanomedicine, we not only preserved MSCs viability but also uncovered a NAD^+^/α‐KG/TET1‐SMPD3 pathway that reprograms exosomal cargo to enrich anti‐inflammatory microRNAs. This dual action, maintaining cell survival while strengthening paracrine immunoregulation, offers a mechanistic resolution to the long‐standing challenge of MSCs instability under chronic inflammation. Importantly, our findings extend the role of NAD^+^ beyond a metabolic safeguard to that of a central regulator linking intracellular energetics with exosome‐mediated immune modulation. These insights provide a strong rationale for metabolic programming as a strategy to achieve more durable and clinically effective MSCs‐based therapies.

## 4 Materials and Methods

### 4.1 Mice and Cell Lines

Female C57BL/6 (H2Kb) mice, aged 6–8 weeks, were obtained from GemPharmatech Co., Ltd. (Jiangsu, China). OT‐I mice were acquired from the Shanghai Model Organisms Center, Inc. (Shanghai, China). All mice were maintained in specific pathogen‐free (SPF) conditions under a 12‐h light/dark cycle, with ad libitum access to food and water. All animal experiments were approved by the Ethical Committee of the Suzhou Institute of Nano‐Tech and Nano‐Bionics, Chinese Academy of Sciences (Protocol No. SINANO/EC/2024‐001). Human MSCs were sourced from Shownin Biotechnology Co., Ltd. (RC02003, China) and cultured in Nuwacell NcMission hMSC Medium V3.0 (RP02010, Shownin) with 100 U mL^−1^ penicillin/streptomycin (C125C5, NCM Biotech) at 37°C in a 5% CO2 incubator. Adherent MSCs were passaged using TrypLE Express (12604013, Thermo Fisher Scientific). RAW264.7 cells were procured from ATCC (Beijing Zhongyuan Ltd.) and cultured in high‐glucose DMEM (C11995500BT, Thermo Fisher Scientific) supplemented with 10% FBS (F8318, Sigma) and 100 U mL^−1^ penicillin/streptomycin at 37°C with 5% CO_2_. Adherent RAW264.7 cells were passaged using 0.25% trypsin‐EDTA (FD7100, Fdbio Science).

### 4.2 Generation of MSCs‐Luc and SMPD3 Knockdown

Lentiviral vectors encoding luciferase and enhanced green fluorescent protein (EGFP) were constructed using the Ubi‐MCS‐firefly luciferase‐3FLAG‐CBh‐gcGFP‐IRES‐puromycin backbone (Genechem, Shanghai, China). MSCs were transduced at an optimized multiplicity of infection and selected with puromycin (5 µg mL^−1^, Solarbio) for 7–10 days. Stable expression of EGFP was verified by fluorescence microscopy. Luciferase activity was quantitatively assessed using a commercial luciferase reporter gene assay kit (11401ES60, YEASEN) and confirmed by luminescence readings. For SMPD3 knockdown, MSCs were transfected with SMPD3‐targeting shRNA cloned into the pLVX‐shRNA2‐Neo vector (Antantares, Jiangsu, China). Transduced cells were selected with G418 (500 µg mL^−1^, Beyotime) for 10–14 days, and knockdown efficiency was validated by quantitative PCR and Western blotting. The shRNA sequences used for SMPD3 knockdown are listed in Table S1.

### 4.3 NANP Preparation

NANP was synthesized using a water‐in‐oil reverse microemulsion technique followed by thin‐film hydration. Briefly, two separate water‐in‐oil reverse microemulsions (25 mL each) were prepared by dispersing IGEPAL CO‐520 (N828401, MACKLIN) in cyclohexane (14224, Adamas) at a volume ratio of 71:29 for the oil phase. Emulsion 1 was composed of 500 µL of 2.5 M CaCl_2_ (015077834, Adamas Life), while Emulsion 2 contained 5 mg NAD^+^ (D0919, TCI), 50 µg PGB, and 0.5 mg DOPA (000502, MeloPEG). The two emulsions were mixed and stirred for 15 min. Ethanol (10 mL) was subsequently added to remove the oil phase, and the nanoparticles were collected by centrifugation at 13,000 × *g* for 30 min. The nanoparticles were washed thrice with ethanol, followed by the addition of 0.6 mg SoyPC (N01003, RVT) and 0.4 mg cholesterol (A90286, Innochem), which were dissolved in chloroform. After chloroform evaporation under rotary evaporation, the lipid film was rehydrated in 500 µL nuclease‐free water to obtain the final NANP formulation.

### 4.4 NANP Characterization

The size distribution and polydispersity index (PDI) were measured using a Malvern NanoSizer (Malvern Instruments Ltd.) in double‐distilled water. NANP morphology was visualized using a Hitachi HT7700 transmission electron microscope (Exalens). The in vitro release kinetics of NAD^+^ from NANP under varying pH conditions were studied using dialysis. NANP were sealed in dialysis bags (MWCO 1000 Da) and immersed in 40 mL buffer at pH 5.0, 6.5, and 7.4. The systems were incubated at 37°C, and 200 µL samples were collected at intervals for NAD^+^ quantification, with fresh prewarmed medium added after each sample. X‐ray powder diffraction (XRD) analysis was carried out using a Bruker AXS D8 Advance diffractometer. The samples were finely ground and loaded into the sample holder. Data were collected across a 2*θ* range of 5° to 90° with a scanning rate of 0.5° per minute. High‐angle annular dark‐field scanning transmission electron microscopy (HAADF‐STEM) and energy dispersive spectroscopy (EDS) mapping were conducted with a Talos F200X microscope and SuperXG2 detector, respectively. Elemental mapping was achieved by capturing and analyzing the characteristic x‐rays emitted from the sample during STEM imaging.

### 4.5 Cell Viability, Apoptotic Experiment, ROS Generation, Mitochondrial Membrane Potential Assays, and HLA‐DR Expression

For cell viability detection, MSCs were seeded at 2000 cells per well in 96‐well plates. They were treated with 10 ng mL^−1^ IFN‐γ (300‐02, PeproTech) plus 10 ng mL^−1^ TNF‐α (300‐01A, PeproTech) alone, or in combination with 25 µM NANP. CCK8 solution (40203ES76, YEASEN) was added at 10 µL per 100 µL culture medium for each well. After a 2‐h incubation at 37°C, the conversion of WST‐8 to formazan by viable cells was measured by optical density (O.D.) at 450 and 600 nm using a microplate reader (Cytation 3, BioTec). For cell death assays, MSCs were seeded at 2 × 10^5^ cells in 12‐well plates. Then 10 ng mL^−1^ IFN‐γ and 10 ng mL^−1^ TNF‐α were added with or without 25 µM NANP for the indicated time. Apoptosis was quantified by dual staining with Annexin V (indicating phosphatidylserine exposure, FMSAV647‐050, FCMACS) and DAPI (2 µg mL^−1^, C1002, Beyotime). For ROS generation, mitochondrial membrane potential and HLA‐DR expression, MSCs were labeled with H2DCFDA (HY‐D0940, MCE), TMRE (C2001S, Beyotime), or HLA‐DR (LN3, Biolegend), respectively. Flow cytometry was used to analyze the data (FACSCelesta, BD Biosciences).

### 4.6 Detection of Cellular NAD(H) Levels and α‐KG Production

MSCs were seeded into 48‐well plates (5 × 10^4^ cells well^−1^) and cultured overnight. Thereafter, the cells were treated with 25 µM free NAD^+^, 25 µM NANP, or IT (10 ng mL^−1^ each) for the indicated time. Following treatment, cells were washed thrice with PBS and lysed in 50 µL lysis buffer. Cellular NAD(H) levels were quantified using the total NAD and NADH Assay Kit (5273, AAT Bioquest), and α‐KG levels were measured using the α‐KG Assay Kit (BC5425, Solarbio), according to the manufacturer's protocols.

### 4.7 Chromatin Immunoprecipitation

Chromatin Immunoprecipitation (ChIP) was performed by using ChIP Assay Kit (P2078, Beyotime) according to the manufacturer's protocol. In brief, cells were crosslinked, and chromatin was extracted and sheared. Immunoprecipitation was performed using anti‐Tet1 antibody (GT1462, GeneTex). Quantitative PCR on the immunoprecipitated DNA was conducted with SYBR Green PCR Master Mix (Q312‐02, Vazyme) and immunoprecipitated DNA values were normalized to input signals.

### 4.8 TET1 Activity Measurement

The Tet enzyme activity was measured using Epigenase 5mC Hydroxylase TET Activity/Inhibition Assay Kit (P‐3086‐48, Epigentek). In brief, MSCs were treated with 25 µM NANP, 1 mM 2‐HG (HY‐113038B, MCE) or 1 mM KG for 24 h. Nuclear and Cytoplasmic Protein Extraction Kit (20126ES50, YEASEN) was used to extract MSCs nuclear protein, the amount of 6 µg protein was used to test TET1 activity.

### 4.9 Murine Inflammatory Bowel Disease Model

Colitis was induced in female C57BL/6J mice by administering 3% DSS (D808272, MACKLIN) in drinking water ad libitum for 8 days. On Day 2 of DSS treatment, mice received an intravenous injection of MSCs that had been pretreated for 4 h with one of the following: 25 µM NANP, an equivalent concentration of Empty NPs, or 25 µM free NAD^+^. In the GW4869 (HY‐19363, MCE) inhibition group, the exosome biogenesis inhibitor was administered intraperitoneally at a dose of 2.5 µg g^−1^ body weight on Day 2 and Day 4 post‐DSS induction. The body weight of each mouse was monitored daily during the experimental period. At the end of the 8‐day DSS treatment, mice were euthanized, and colon tissues were collected for further analysis. Serum IL‐6 levels were measured using a mouse IL‐6 ELISA kit (SEK50135, Sino Biological Inc.). Disease activity was assessed by evaluating body weight loss (graded 0–4), stool consistency (graded 0–2), rectal bleeding (graded 0–2), and general activity (graded 0–2).

### 4.10 Histopathological Analysis

Colon tissues were fixed in 4% paraformaldehyde for 2 days, followed by progressive dehydration and paraffin embedding. Tissue sections (3 µm) were stained with hematoxylin and eosin (H&E). The severity of inflammation was assessed by scoring the degree of bowel wall thickening (graded 0–3), crypt damage (graded 0–3), and inflammatory cell infiltration (graded 0–2).

### 4.11 Analyses of Immune Cell Proportion in the Colitic Tissue

Colon samples were excised, fat removed, and rinsed in PBS. They were cut into 1 cm pieces and incubated in warm HBSS (C0218, Beyotime) with 5% FBS, 2 mM DTT (ST043, Beyotime), and 5 mM EDTA (E8040, Solarbio) at 37°C for 20 min on a shaker. The tissue was then digested in DMEM with 10% FBS, collagenase I (1 mg mL^−1^, BS163, Biosharp), and dispase II (11 U mL^−1^, 40104ES60, YEASEN) at 37°C for 1 h. The digested tissue was filtered through a 70‐µm cell strainer to obtain a single‐cell suspension. Cells were stained for Zombie Yellow Fixable Viability Kit (423103, BioLegend), CD45 (I3/2.3, eBioscience), CD11b (M1/70, Tonbo), Ly6G (1A8, Tonbo), F4/80 (BM8, BioLegend), CD206 (CD68C2, BioLegend), CD11c (N418, BioLegend), IA/IE (M5/114.15.2, BioLegend), CD3 (17A2, BioLegend), CD4 (RM4‐5, Tonbo), CD8 (53‐6.7, Biolegend), NK1.1 (PK136, Biolegend), and CD19 (HIB19, eBioscience) and analyzed by flow cytometry using FlowJo.

### 4.12 In Vivo Bioluminescent and Fluorescence Imaging

Bioluminescent imaging (BLI) and fluorescence imaging were used to evaluate the survival and distribution of MSCs in vivo. For BLI, 1 × 10^6^ MSCs‐Luc were injected intraperitoneally into mice with 2 mg kg^−1^ LPS‐induced inflammation. Ten minutes prior to imaging, D‐luciferin potassium salt (450 mg kg^−1^; 011471829, Adamas) was administered intraperitoneally. Imaging was performed once the bioluminescent signal reached a stable plateau. For fluorescence imaging, MSCs were labeled with 10 µM DiR iodide (40757ES25, YEASEN) according to the manufacturer's protocol, which enabled tracking of MSCs in tissues with enhanced fluorescence intensity. Forty‐eight hours after MSCs‐Luc injection, the mice were euthanized, and the colon was extracted. Both bioluminescent and fluorescence signals were captured and analyzed using the IVIS Lumina III Small Animal Imaging System (PerkinElmer).

### 4.13 Western Blotting Analysis and Quantitative PCR

Cell pellets were resuspended in lysis buffer (P0013, Beyotime), supplemented with 1 mM phenylmethylsulfonyl fluoride (PMSF, ST505, Beyotime), and subjected to sonication. Following lysis, protein samples were denatured by boiling and resolved by SDS‐PAGE using 12.5% polyacrylamide gels (PG112, EpiZyme) in Tris/Glycine/SDS running buffer (G2152, Servicebio). The separated proteins were transferred onto polyvinylidene fluoride (PVDF) membranes (FFP39, Beyotime) and blocked with 5% bovine serum albumin (BSA, BS114, Biosharp) at room temperature for 1 h. Membranes were incubated with primary antibodies (diluted 1:1000) overnight at 4°C, followed by incubation with horseradish peroxidase (HRP)‐conjugated secondary antibodies (1:3000, room temperature, 1 h). The primary antibodies used included CD81 (1.3.3.22), CD63 (E‐12), TSG101 (C‐2), and ALIX (1A12) from Santa Cruz Biotechnology; N‐SMase2/SMPD3 (PA5‐117447) from Thermo Fisher Scientific; TET1 (GT1462) from GeneTex; CASPASE‐3 (14220), cleaved CASPASE‐3 (9661), RIP3 (10188), p‐RIP3 (93654), and PD‐L1 (E1L3N) from Cell Signaling Technology; MLKL (ab184718) and p‐MLKL (ab187091) from Abcam; and ACTIN (HRP‐60008) from Proteintech. The HRP‐conjugated secondary antibodies used were anti‐rabbit IgG (7074) and anti‐mouse IgG (7076) from Cell Signaling Technology. Protein bands were visualized using an enhanced chemiluminescence (ECL) substrate kit (36222ES60, YEASEN) and detected with an Amersham Imager 600 (Cytiva). Quantitative RT‐PCR was performed by reverse transcription of 500 ng RNA (R223‐01, Vazyme), followed by SYBR Green‐based real‐time PCR assays (Q312‐02, Vazyme). All primers were designed using Primer5 (see Table S4). For the miRNA analysis of MSCs‐derived exosomes, cDNAs of mRNAs were synthesized using the Hifair miRNA first Strand cDNA Synthesis Kit (11148ES10, YEASEN). The expression levels of target miRNAs were quantified using SYBR reagent (11171ES, YEASEN), with U6 serving as the reference gene. Both assays were performed on the Q2000B fluorescent quantitative PCR system (LongGene, China). Fold changes in expression were calculated using the comparative Ct algorithm ($2^{-\Delta \Delta C_{t}}$).

### 4.14 Immunofluorescence Staining

To evaluate NANP uptake, MSCs were seeded overnight in 35 mm culture dishes. 25 µM NANP was labeled with the 10 µM DiD probe (C1039, Beyotime) and added to the MSCs culture medium. After 4 h of incubation, cells were co‐stained with 100 nM LysoTracker Green (C1047S, Beyotime) and 0.1 mg mL^−1^ Hoechst 33342 (C1022, Beyotime) for 15 min at 37°C.  For assessing the uptake of MSCs‐exo, 20 µg mL^−1^ DiO‐labeled MSCs‐exo were incubated with RAW264.7 macrophages or BMDCs for 4 h, followed by analysis using immunofluorescence microscopy or flow cytometry. For ex vivo colon tissue staining, distal colon sections were harvested from mice, fixed in 4% paraformaldehyde (BL539A, Biosharp), and embedded in optimal cutting temperature compound before freezing. Cryosections (8 µm) were washed with cold PBS and blocked with 10% BSA and 0.3% Triton X‐100 for 1 h at room temperature. Sections were then incubated overnight at 4°C with primary antibodies against E‐cadherin (sc‐52327, Santa Cruz Biotechnology), F4/80 (28463‐1‐AP, Proteintech), and CD206 (8704‐1‐AP, Proteintech). Subsequently, the tissue sections were performed on the TYR‐520/570/690 Fluorescent Kit (AFIHC026, AiFang biological, China). Fluorescent images were captured using a confocal microscope (FV3000, Olympus).

### 4.15 Exosome Isolation and Characterization

MSCs culture medium was first centrifuged at 10,000 × *g* for 30 min to remove cellular debris, followed by filtration through a 0.2 µm filter. The supernatant was then subjected to ultracentrifugation at 120,000 × *g* for 70 min at 4°C. The resulting exosomes pellet was washed in PBS (centrifuged again at 120,000 × *g* for 70 min) and resuspended in nuclease‐free water. Exosome size and concentration using NTA (NS300, Malvern). For TEM, exosomes were fixed with 2% paraformaldehyde and adsorbed onto Formvar‐ and carbon‐coated copper grids. The grids were incubated with 2% gelatin at 37°C for 20 min, rinsed with 0.15 M glycine/PBS, and blocked with 1% cold‐water fish‐skin gelatin. Exosomes morphology was visualized using a Hitachi HT7700 transmission electron microscope (Exalens). For in vitro experiments, exosomes were applied at a concentration of 20 µg mL^−1^ based on protein content, which was determined using the BCA Protein Assay Kit (WB6501, HCN Biotech).

### 4.16 Preparation of Different MSCs‐Exo

MSCs‐exo were derived from MSCs cultured under normal conditions. To obtain exosomes from MSCs treated with inflammatory stimuli (IT MSCs‐exo), MSCs were exposed to IT (10 ng mL^−1^ each) for 48 h, and exosomes were collected from the conditioned medium. For NAD^+^‐loaded MSCs‐exo, MSCs were treated with 25 µM NANP for 4 h. The medium was then replaced with fresh complete culture medium, and the conditioned medium was collected after an additional 48 h. To prepare exosomes from MSCs treated with both inflammatory stimuli and NANP (IT+NAD^+^‐loaded MSCs‐exo), MSCs were first treated with 25 µM NANP for 4 h. Subsequently, the medium was replaced with fresh medium containing IT (10 ng mL^−1^ each), and the conditioned medium was collected after an additional 48 h.

### 4.17 MiRNA Mimic, miRNA Inhibitors, or siRNA Transfection

Recipient cells were transfected with 100 nM miRNA mimic, miRNA inhibitors or siRNA (both from Antantares, China) using the GP‐Transfect‐Mate transfection reagent (230704, GenePharma) according to the manufacturer's instructions. The transfection mixture was prepared in serum‐free medium and incubated with cells for 4–6 h, followed by replacement with complete growth medium. Transfection efficiency was assessed 24 h later by quantitative PCR to measure targeted mRNA expression. The siRNA sequences used for TET1 knockdown are listed in Table S2, and the sequences of the miRNA mimics and inhibitors are provided in Table S3.

### 4.18 RNA Sequencing

For mRNA sequencing, MSCs were treated with 25 µM NANP for 4 h. Exosomes and cells were collected by replacing the supernatant and continuing the culture for an additional 48 h. Subsequently, 1 µg of total RNA from each sample was used for RNA sequencing analysis. Exosomal small RNA sequencing was performed with 20 ng of RNA per sample. Both sequencing libraries were prepared and processed by Azenta Life Sciences Co., Ltd. (Jiangsu, China). Differential expression analysis was conducted using the DESeq2 Bioconductor package, which is based on a negative binomial distribution model. Dispersion estimates and log2 fold changes were computed using data‐driven prior distributions, with genes having an adjusted p‐value (Padj) ≤ 0.05 considered differentially expressed. Gene Ontology (GO) term enrichment was performed using GOSeq (v1.34.1) to annotate enriched genes, with a significance threshold of Padj ≤ 0.05.

### 4.19 BMDC Generation and Maturation

Bone marrow cells were isolated from female C57BL/6 mice and subjected to red blood cell lysis using RBC lysis buffer (C3702, Beyotime). The remaining cells were cultured in RPMI‐1640 medium supplemented with 20 ng mL^−1^ granulocyte macrophage colony stimulating factor (GM‐CSF; AF‐315‐03, PeproTech) and 10 ng mL^−1^ interleukin‐4 (IL‐4; 214‐14, PeproTech) for 6 days. On Day 6, BMDC were treated with 40 µg mL^−1^ Poly(I:C) (P2425, Regent Science Industry) plus 20 µg mL^−1^ MSCs‐exo or NAD^+^‐loaded MSCs‐exo for 24 h. BMDC maturation was assessed by flow cytometry based on the expression of CD86 (B7‐2, eBioscience) and MHC‐II (M5/114.15.2, BioLegend).

### 4.20 CD8^+^ T Cell Priming Assay

For OT‐I proliferation, BMDC were pulsed overnight with 0.1 mg mL^−1^ ovalbumin (OVA; A5503, Sigma). OT‐I cells were isolated from OT‐I transgenic mouse spleens using the Mouse CD8^+^ T Cell Isolation Kit (CS103‐01, Vazyme) and labeled with eFluor 670 dye (65‐0840‐85, eBioscience). These OT‐I cells (1 × 10^5^ cells well^−1^) were co‐cultured with 2 × 10^4^ OVA‐pulsed BMDC in the presence or absence of 20 ng mL^−1^ MSCs‐exo for 72 h. For anti‐CD3/CD28‐stimulated CD8^+^ T cell expansion, sterile 96‐well flat‐bottom plates were coated overnight at 4°C with 2 µg mL^−1^ anti‐CD3 (145‐2C11, BioLegend) and 0.4 µg mL^−1^ anti‐CD28 (102116, BioLegend) in PBS. Purified CD8^+^ T cells (1  ×  10^5^ cells well^−1^) from C57BL/6 mice were labeled with eFluor 670 dye and then co‐cultured with 1  ×  10^4^ MSCs in the coated wells for 72 h. MSCs in the treatment groups were pretreated as follows: IT‐treated MSCs were stimulated with 10 ng mL^−1^ IFN‐γ and 10 ng mL^−1^ TNF‐α for either 24 h (1d IT treatment) or 5 days (5d IT treatment); FK866‐treated MSCs were exposed to 20 nM FK866 (HY‐50876, MCE) for 24 h. In all assays, CD8^+^ T cell proliferation was assessed by eFluor 670 dye dilution using flow cytometry on a FACSCelesta system (BD Biosciences).

### 4.21 Polarization of M1‐ and M2‐Like Macrophages

To examine macrophage polarization, RAW264.7 cells were initially stimulated with 100 ng mL^−1^ LPS (916374, Sigma) and 10 ng mL^−1^ IFN‐γ (315‐05, PeproTech) for 2 h to induce M1‐like polarization. Following this initial stimulation, the culture medium was replaced with exosomes‐enriched fractions derived from MSCs or NAD^+^‐loaded MSCs for 12 h. The polarization status of macrophages was assessed by flow cytometry, analyzing the frequencies of M1‐ (iNOS, 14‐5920‐82, Thermo Fisher Scientific) and M2‐ (CD206, sc‐58986, Santa Cruz Biotechnology) like macrophage populations. Additionally, the expression levels of *Tnf‐α*, *Nos2*, *Il‐6* and *Il‐10* were quantified using quantitative PCR to confirm the polarization phenotype.

### 4.22 Statistical Analysis

All experimental groups were randomized to eliminate bias. Data were analyzed using GraphPad Prism 8 software. For comparisons between two groups with normal distribution, a two‐tailed unpaired Student's *t*‐test was employed. For non‐normally distributed data, the Mann–Whitney U test was used. One‐way ANOVA was applied for comparisons among three or more groups. Data are expressed as mean ± SEM. Statistical significance was determined with *p < 0.05, **p < 0.01, and ***p < 0.001.

## Author Contributions

Mingzhou Ye and Jinfeng Chen. Investigation: Jinfeng Chen, Kanghua Zeng, Jinhong Chao, Xiangna Zhang, Zheng Zhou, and Xiang Li. Formal analysis: Jinfeng Chen, Kanghua Zeng, Jinhong Chao, Wancheng Liu, Xiahong Xie, and Luyao Wang. Writing: Jinfeng Chen and Mingzhou Ye. Review and editing: All authors. All authors reviewed and approved the final version of the manuscript.

## Conflicts of Interest

The authors declare no conflicts of interest.

## Ethics Statement

All animal experiments were approved by the Ethical Committee of the Suzhou Institute of Nano‐Tech and Nano‐Bionics, Chinese Academy of Sciences (Protocol No. SINANO/EC/2024‐001), and were conducted in accordance with the approved institutional guidelines.

## Declaration of Generative AI and AI‐Assisted Technologies in the Writing Process

During the preparation of this manuscript, the authors used ChatGPT (OpenAI) for English language editing and stylistic refinement in the Abstract, Introduction, Results, Discussion, and figure legends. ChatGPT was not used for data generation or analysis, or for preparing figures, tables, supplementary data, or raw data. All AI‐assisted text was reviewed and verified by the authors.

## Supporting information




**Supporting File 1**: exp270249‐sup‐0001‐SuppMat.docx.

**Supporting File 2**: exp270249‐sup‐0002‐SuppMat_Tables_S1‐S4.docx.

## Data Availability

All raw RNA sequencing data can be found at the Gene Expression Omnibus (GEO) using the accession code GSE286990. The data that support the findings of this study are available from the corresponding author upon reasonable request.
