# Supplementary material for: Metabolic Nanomedicine Reprograms Mesenchymal Stem Cells for Durable Exosome‐Mediated Immunomodulation
Source: Exploration (Beijing). 2026 Nov 10:70249. Online ahead of print. doi: 10.1002/exp2.70249 (PMC13752232; doi:10.1002/exp2.70249)
Supplement: Supplementary file 1 [file EXP2-9999-0-s001.docx]

**Table S1. Sequence of shRNA for SMPD3 knockdown.**

| Gene | Sequence 5’-3’ |
| --- | --- |
| *Smpd3* | CCGGCGCATCGACTACATGCTGCATCTCGAGATGCAGCATGTAGTCGATGCGTTTTTT |
| *Ctrl* | CCGGTTCTCCGAACGTGTCACGTTTCTCGAGACGTGACACGTTCGGAGAATTTTTTTG |

**Table S2. Sequence of siRNA for TET1 knockdown.**

| siRNA | Forward | Reverse |
| --- | --- | --- |
| *Tet1* | GGAUAGAAAUAGUAGUGUACA | UACACUACUAUUUCUAUCCUU |
| *siRNA-ctrl* | UUCUCCGAACGUGUCACGUdTdT | ACGUGACACGUUCGGAGAAdTdT |

**Table S3. miRNA mimics and inhibitors**

| miRNA | Forward | Reverse |
| --- | --- | --- |
| *hsa-miR-146a-5p* | UGAGAACUGAAUUCCAUGGGUU | CCCAUGGAAUUCAGUUCUCAUU |
| *hsa-miR-146b-5p* | UGAGAACUGAAUUCCAUAGGCUG | GCCUAUGGAAUUCAGUUCUCAUU |
| *hsa-miR-30a-5p* | UGUAAACAUCCUCGACUGGAAG | UCCAGUCGAGGAUGUUUACAUU |
| *miRNA-ctrl* | UUCUCCGAACGUGUCACGUTT | ACGUGACACGUUCGGAGAATT |
| *hsa-miR-146a-5p*-inhibitor | /i2OMeA//i2OMeA//i2OMeC//i2OMeC//i2OMeC//i2OMeA//i2OMeU//i2OMeG//i2OMeG//i2OMeA//i2OMeA//i2OMeU//i2OMeU//i2OMeC//i2OMeA//i2OMeG//i2OMeU//i2OMeU//i2OMeC//i2OMeU//i2OMeC//i2OMeA/ | |
| *hsa-miR-146b-5p*-inhibitor | /i2OMeC//i2OMeA//i2OMeG//i2OMeC//i2OMeC//i2OMeU//i2OMeA//i2OMeU//i2OMeG//i2OMeG//i2OMeA//i2OMeA//i2OMeU//i2OMeU//i2OMeC//i2OMeA//i2OMeG//i2OMeU//i2OMeU//i2OMeC//i2OMeU//i2OMeC//i2OMeA/ | |
| *hsa-miR-30a-5p*-inhibitor | /i2OMeC//i2OMeU//i2OMeU//i2OMeC//i2OMeC//i2OMeA//i2OMeG//i2OMeU//i2OMeC//i2OMeG//i2OMeA//i2OMeG//i2OMeG//i2OMeA//i2OMeU//i2OMeG//i2OMeU//i2OMeU//i2OMeU//i2OMeA//i2OMeC//i2OMeA/ | |
| *miR-ctrl*-inhibitor | /i2OMeC//i2OMeA//i2OMeG//i2OMeU//i2OMeA//i2OMeC//i2OMeU//i2OMeU//i2OMeU//i2OMeU//i2OMeG//i2OMeU//i2OMeG//i2OMeU//i2OMeA//i2OMeG//i2OMeU//i2OMeA//i2OMeC//i2OMeA//i2OMeA/ | |

**Table S4. Primers for qRT-PCR**

| Gene | Forward | Reverse |
| --- | --- | --- |
| H*-Gapdh* | GTCTCCTCTGACTTCAACAGCG | ACCACCCTGTTGCTGTAGCCAA |
| H*-Smpd3* | GCTGCCCTTTGCGTTTCTC | TCCAGCCGTGAATAGATGTAGG |
| H*-Ybx1* | GGGGACAAGAAGGTCATCGC | CGAAGGTACTTCCTGGGGTTA |
| H*-Syncrip* | CTGGTCTCAATAGAGGTTATGCG | TCCGGTTGGTGGTATAAAATGAC |
| H*-Hnrnpa2b1* | ATTGATGGGAGAGTAGTTGAGCC | AATTCCGCCAACAAACAGCTT |
| H*-Anxa2* | TCTACTGTTCACGAAATCCTGTG | AGTATAGGCTTTGACAGACCCAT |
| H*-Tet1* | CATCAGTCAAGACTTTAAGCCCT | CGGGTGGTTTAGGTTCTGTTT |
| H*-Pd-l1* | TGGCATTTGCTGAACGCATTT | TGCAGCCAGGTCTAATTGTTTT |
| H*-Il-10* | GACTTTAAGGGTTACCTGGGTTG | TCACATGCGCCTTGATGTCTG |
| H*-Il-4* | CCAACTGCTTCCCCCTCTG | TCTGTTACGGTCAACTCGGTG |
| H*-Tsg-6* | TTTCTCTTGCTATGGGAAGACAC | GAGCTTGTATTTGCCAGACCG |
| H*-Ido* | GCCAGCTTCGAGAAAGAGTTG | ATCCCAGAACTAGACGTGCAA |
| H*-Pge2* | CGATGCTCATGCTCTCTTCGC | GGGAGACTGCATAGATGACAGG |
| H*-Tgf-β* | GGCCAGATCCTGTCCAAGC | GTGGGTTTCCACCATTAGCAC |
| H-*Sirt1* | AAGTTGACTGTGAAGCTGTACG | TGCTACTGGTCTTACTTTGAGGG |
| M-*Ppia* | GAGCTGTTTGCAGACAAAGTTC | CCCTGGCACATGAATCCTGG |
| M-*Tnf-a* | GACGTGGAACTGGCAGAAGAG | TTGGTGGTTTGTGAGTGTGAG |
| M-*Il-6* | TAGTCCTTCCTACCCCAATTTCC | TTGGTCCTTAGCCACTCCTTC |
| M-*Nos2* | GTTCTCAGCCCAACAATACAAGA | GTGGACGGGTCGATGTCAC |
| M-*Il-10* | GCTCTTACTGACTGGCATGAG | CGCAGCTCTAGGAGCATGTG |
| *hsa-miR-146a-5p* | TGAGAACTGAATTCCATGGGTT | Universal Reverse Primer provided by YEASEN (Cat#11148) |
| *hsa-miR-146b-5p* | TGAGAACTGAATTCCATAGGCTG |  |
| *hsa-miR-30a-5p* | TGTAAACATCCTCGACTGGAAG |  |
