# Supplementary material for: Metabolic Nanomedicine Reprograms Mesenchymal Stem Cells for Durable Exosome‐Mediated Immunomodulation
Source: Exploration (Beijing). 2026 Nov 10:70249. Online ahead of print. doi: 10.1002/exp2.70249 (PMC13752232; doi:10.1002/exp2.70249)
Supplement: Supplementary file 2 [file EXP2-9999-0-s002.docx]

**Supplementary information**

**FIGURE S1. The vitality and anti-inflammatory regulatory functions of MSCs impaired by inflammation.** (**A**) Flow cytometry analysis of EGFP expression in MSCs-Luc versus MSCs, confirming successful cell construction. (**B**) Relative Light unit (RLU) of luciferase activity in MSCs-Luc compared to MSCs (n = 5). (**C**) Schematic of the in vivo imaging protocol. (**D**) In vivo tracking of intraperitoneally injected MSCs-Luc at the indicated time points (n = 3). (**E**) Comparison of the average radiance of MSCs-Luc between PBS-treated and 2 mg kg^-1^ LPS-treated mice (n = 7). (**F-G**) IT (10 ng mL^-1^ each) induced cell death at 24 h (F) and 48 h (G), quantified by Annexin V/DAPI staining. Representative cytofluorometric dot plots and statistical analyses are shown (n = 3). (**H**) MSCs were pretreated with IT (10 ng mL^-1^ each) for 1 day (1d IT‑MSCs) or 5 days (5d IT‑MSCs), or with 20 nM FK866 for 24 h (FK866‑MSCs), before being co‑cultured with stimulated CD8⁺ T cells. Representative flow cytometric dot plots and statistical analyses are shown (n = 3). All data were shown as mean ± SEM. * p < 0.05, ** p < 0.01, *** p < 0.001.

**FIGURE S2. NANP alleviates inflammation-induced MSCs death and promotes IBD therapy.** (**A**) Flow cytometry analysis of IT (10 ng mL^-1^ each, 48 h) induced ROS generation and its inhibition by NANP. Representative histograms and statistical analysis of mean fluorescence intensity (MFI) are shown (n = 3). (**B**) Flow cytometry analysis of HLA-DR expression on MSCs stimulated with IT (10 ng mL^-1^, each, 48 h) (left), and the quantification of the percentage of HLA-DR positive MSCs (right) (n = 3). (**C**) MSCs were treated with 25 μM NANP, IT, or their combination for 48 h. The expression of necroptosis executors (MLKL and p-MLKL) was assessed by semi-quantitative Western blotting. (**D**)  Body weight changes of IBD mice treated with MSCs, Free NAD⁺-treated MSCs, Empty NPs-treated MSCs or NAD^+^-loaded MSCs (n = 5). (**E-F**) Colon length of IBD mice and its quantitative analysis across groups treated with MSCs, Free NAD⁺-treated MSCs, Empty NPs-treated MSCs or NAD^+^-loaded MSCs (n = 5). (**G**) Disease activity index of IBD mice treated with MSCs, Free NAD^+^-treated MSCs, Empty NPs-treated MSCs or NAD^+^-loaded MSCs (n = 5). (**H**) Disease activity index of IBD mice treated with MSCs or NAD^+^-loaded MSCs (n = 5). (**I**) Interleukin-6 (IL-6) levels in the serum of IBD mice treated with MSCs or NAD^+^-loaded MSCs were assayed by enzyme-linked immunosorbent assay (ELISA) (n = 5). (**J**) Representative immunofluorescence image of E-Cadherin (Green) and Nuclei (blue) in colon tissue from MSCs or NAD^+^-loaded MSCs treated IBD mice. (**K**) In vivo distribution of DiR‑labeled MSCs in IBD mice. Quantitative analysis of average radiant efficiency in the lung, liver, spleen, heart, kidney, and lymph nodes from mice receiving MSCs or NAD⁺-loaded MSCs. (**L**) Serum alanine aminotransferase (ALT) and aspartate aminotransferase (AST) levels in mice from the MSCs and NAD⁺-loaded MSCs groups (n = 3). All data were shown as mean ± SEM. * p < 0.05, ** p < 0.01, *** p < 0.001.

**FIGURE S3. NANP-based MSCs augment local colon immunological tolerance.** (**A-B**) Cytofluorometric plots display the gating strategies for immune cell subsets. (**C**) Gating strategy for identifying F4/80^+^ macrophages. (**D**) Representative immunofluorescence image showing F4/80 (green), CD206 (red), and nuclei (blue) in colon tissue from IBD mice treated with MSCs or NAD^+^-loaded MSCs. (**E**) Typical image of M2 macrophages from NAD^+^-loaded MSCs group.

**FIGURE S4. Characterization and functional assessment of MSCs-derived exosomes.** (**A**)  Body weight changes of IBD mice treated with NAD⁺-loaded MSCs, with or without GW4869 co‑administration (n = 5). (**B**)  Representative images of colon length from IBD mice treated with NAD⁺-loaded MSCs, with or without GW4869 (n = 5). (**C**) Quantitative analysis of colon length and disease activity index (DAI) in IBD mice treated with NAD⁺-loaded MSCs, with or without GW4869 (n = 5). (**D**) Schematic diagram showing the preparation of different MSCs-exo regimen. (**E**) Western blot analysis of the exosomal markers ALG-2-interacting protein X (Alix), Tumor susceptibility gene 101 (TSG101), Cluster of Differentiation 81 (CD81) and Cluster of Differentiation 63 (CD63). (**F**) Size distribution and transmission electron microscopy (TEM) image of exosomes derived from MSCs or MSCs treated with NANP. (**G**) Particle concentrations of MSCs-exo or NAD^+^-loaded MSCs-exo through nanoparticle tracking analysis (n = 3). (**H**) Immunofluorescence imaging of RAW264.7 cells following uptake of DiO‑labeled MSCs-exo, showing enhanced intracellular fluorescence. (**I**) Flow cytometry analysis confirms enhanced mean fluorescence intensity in RAW264.7 cells and BMDC following internalization of DiO‑labeled MSCs-exo (n = 3). (**J**)  RAW264.7 cells were pretreated with LPS (100 ng mL^-1^) and IFN‑γ (10 ng mL^-1^) for 2 h, followed by incubation with exosomes (20 µg mL^-1^) from the following sources for an additional 12 h: untreated MSCs, free NAD⁺‑treated MSCs, Empty NPs-treated MSCs, or NAD⁺‑loaded MSCs. The mRNA levels of *Tnf‑α*, *Il-6*, and *Nos2* were subsequently quantified by qRT‑PCR (n = 3). All data were shown as mean ± SEM. * p < 0.05, ** p < 0.01, *** p < 0.001.

**FIGURE S5.** **MSCs-sh-Smpd3 exhibit reduced immunomodulatory responsiveness to NANP and miRNA-mediated effects.** (**A**) Heatmap analysis of down-regulated differential miRNA among NAD^+^-loaded MSCs-exo and MSCs-exo. (**B**) qRT-PCR analysis of miR-30a-5p, miR-146a-5p, and miR-146b-5p expression in RAW264.7 and BMDC with MSCs-exo or NAD^+^-loaded MSCs-exo (n = 3). (**C**) In RAW264.7 cells stimulated with LPS (100 ng mL^-1^) and IFN-γ (10 ng mL^-1^), NAD⁺‑loaded MSCs‑exo suppressed the up‑regulated expression of pro‑inflammatory mediators (*Tnf‑α*, *Il‑6*, and *Nos2*). This anti‑inflammatory effect was reversed by co‑transfection with a miR‑30a/146a/146b inhibitor cocktail (n = 3). (**D**) In Poly(I:C)-stimulated BMDC, NAD⁺‑loaded MSCs‑exo inhibited the up‑regulation of the surface markers CD86 and MHC‑II. This inhibition was abrogated by co‑transfection with miR‑30a/146a/146b inhibitors (n=3). (**E**) Size distribution profiles of exosomes derived from MSCs‑sh‑Ctrl and MSCs‑sh‑SMPD3, with or without NANP treatment (left panel), and corresponding particle concentrations determined by nanoparticle tracking analysis (right panel) (n = 3). All data were shown as mean ± SEM. * p < 0.05, ** p < 0.01, *** p < 0.001.

**FIGURE S6. TET1 promotes the expression of SMPD3, thereby inducing colonic immune tolerance.** (**A**) RAW264.7 cells were pretreated with LPS (100 ng mL^-1^) and IFN-γ (10 ng mL^-1^) for 2 h, then cultured with 20 µg mL^-1^ exosome from MSCs-sh-Ctrl or MSCs-sh-Smpd3 for another 12 h. The mRNA expressions of *Tnf-a, Il-6* and *Nos2* genes were measured by qRT-PCR (n=3). (**B**) Representative histograms illustrating CD86 and MHC-II expression in BMDC after Poly(I:C) stimulation with or without different MSCs-exo. Statistical analysis of mean fluorescence intensity (MFI) is shown (n = 3). (**C**) Disease activity index of IBD mice treated with MSCs-sh-Ctrl or MSCs-sh-Smpd3, with or without NANP (25 µM, 4 h) treatment (n = 4-5). (**D**) Proportion of major colonic immune cell subsets quantified by flow cytometry (n = 6). (**E**) The mRNA expression levels of *Smpd3* in MSCs treated with 25 µM NANP, 10 µM NAM or the combination for 24 h (n = 3). (**F**) The CpG island of *Smpd3* promoter. (**G**) TET1 binding on the promoter of *Smpd3* in MSCs, as assessed by ChIP-qPCR, IgG was used as a control (n = 5). All data were shown as mean ± SEM. * p < 0.05, ** p < 0.01, *** p < 0.001.
